# Bridging Molecular and Bulk Nonlinearities: Kerr Effect Phenomena in Transparent Ceramic Systems

**DOI:** 10.3390/ijms27031352

**Published:** 2026-01-29

**Authors:** Andrzej Kruk, Mateusz Schabikowski

**Affiliations:** 1 AGH University of Kraków, Faculty of Space Technologies, al. A. Mickiewicza 30, 30-059 Kraków, Poland; 2 Institute of Nuclear Physics Polish Academy of Sciences, Radzikowskiego 152, 31-342 Kraków, Poland; mateusz.schabikowski@ifj.edu.pl

**Keywords:** Kerr effect, transparent ceramics, magneto-optic, electro-optic, molecular spectroscopy

## Abstract

Transparent ceramics offer a uniquely accessible platform for examining Kerr-type optical phenomena through the lens of molecular structure and local electronic interactions. This review highlights both the magneto-optical (MOKE) and electro-optic (EOKE) forms of the Kerr effect and relates them to the accompanying Faraday and Cotton–Mouton responses. We briefly outline material classes exhibiting Kerr activity—from classic spinels and garnets to perovskites and modern composite ceramics. Particular attention is given to the molecular and atomic mechanisms underlying Kerr behavior—crystal symmetry, site-specific ionic coordination, covalency, electronic-level splitting, carrier localization, vacancy chemistry, and the influence of dopants on polarizability and nonlinear susceptibility. We also summarize advances in experimental setups that have improved measurement precision and spectral range. Selected examples demonstrate how molecular-scale control over electronic structure enables diverse and tunable Kerr responses in different ceramics. We conclude by identifying key remaining challenges in materials design and measurement techniques, and by pointing to future directions driven by improved synthesis and molecular-level engineering.

## 1. Introduction

Historically, glass was a predominant transparent material which has been manufactured by mankind as early as the second millennium BC [1]. The process of manufacture improved through the ages, and the material became progressively clearer and more transparent. In the XIX century, celluloid and Bakelite®emerged as the modern materials and the first plastics ever created [2]. The XX century gave rise to more advanced transparent materials like polymethyl methacrylate, polycarbonate through optical fibers (silica glass fibers) to the first sintered transparent ceramics such as MgAl_2_O_4_ spinel, aluminum oxynitride ((AlN)_*x*_·(Al_2_O_3_)_1−*x*_, ALON), and yttrium aluminum garnet (Y_3_Al_5_O_12_, YAG).

Since the earliest scientific discoveries concerning the interactions between matter and external fields—such as magnetic, electric, and electromagnetic (EM) fields—research devoted to understanding these effects has gained increasing importance. Comprehending the behavior of solids, and molecules in particular, lies at the foundation of modern science and experimental research. The interactions between molecules and external fields are also highly relevant from an applied perspective. Properly designed magnetic fields can be used to orient or manipulate molecular systems. Electric fields induce molecular polarization by redistributing charge within the molecule. Electromagnetic fields, in turn, can alter the internal energy of molecules by driving rotational, vibrational, or electronic transitions. Thus, depending on the type and strength of the applied external field, molecules exhibit characteristic behaviors that can be directly observed through various physical properties. Understanding these responses not only deepens our knowledge of fundamental molecular physics but also enables practical applications in spectroscopy, material design, and the control of chemical processes. Even more important and intriguing phenomena in molecules can be observed when the interaction of an electromagnetic field is combined with an applied magnetic or electric field. Such field–EM field–matter interactions often lead to nonlinear effects and reveal subtle aspects of molecular structure and dynamics that are not accessible under a single external field.

In 1845, Michael Faraday discovered a phenomenon in solid-state materials in which the plane of polarization of linearly polarized light is rotated as it passes through a sample subjected to a magnetic field aligned with the direction of light propagation. This observation was historically significant, as it provided the first experimental evidence of an interaction between light and a magnetic field. It was not until the late 1860s and early 1870s that J. C. Maxwell established the theoretical foundation for this effect, now known as the Faraday effect or magneto-optical rotation. Although originally observed in bulk, optically inactive solids, the Faraday effect can also be understood at the molecular level. When linearly polarized light travels parallel to the magnetic field vector B, the field perturbs the electronic motion within molecules, lifting degeneracies and modifying the refractive indices for left- and right-circularly polarized components of the light. The resulting difference in phase velocities leads to a rotation of the polarization plane. This molecular interpretation highlights the Faraday effect as an important contribution to the broader study of light–matter interactions, revealing how external magnetic fields can influence molecular electronic structure and optical response.

The electrical Kerr effect was first discovered in 1875 by the Scottish physicist John Kerr. In essence, during his initial investigations into how light interacts with magnetic materials, Kerr discovered that the polarization of a reflected light beam was altered when plane-polarized light was directed onto the surface of a horseshoe magnet. The change in the optical properties of a crystal due to the action of an electric field on the crystal is called the electro-optic phenomenon. The Kerr effect is a third-order non-linear optical process in which the refractive index of a medium is modified by an applied electric field. This field can be either static (generated by a capacitor) leading to the electro-optic Kerr effect (the electrical Kerr effect), or it may be the electric field component of an intense electromagnetic wave—resulting in the optical Kerr effect (the optical–optical Kerr effect). Additionally, there exists the magneto-optic Kerr effect, where the refractive index is altered under the influence of a magnetic field. From a molecular standpoint, these Kerr effects arise because external fields perturb the electronic charge distribution within molecules, inducing field-dependent changes in molecular polarizability and higher-order hyperpolarizabilities. In anisotropic or partially ordered systems, such perturbations lead to field-induced birefringence—the hallmark of Kerr-type responses. Thus, the Kerr effects not only play a fundamental role in nonlinear optics but also provide valuable insight into molecular electronic structure and its susceptibility to external fields.

The Cotton–Mouton effect is a magneto-optical phenomenon in which birefringence is induced in a transparent, initially isotropic medium under the influence of an external magnetic field applied perpendicular to the direction of light propagation. First identified in 1905 by French physicists Aimé Cotton and Henri Mouton, the Cotton–Mouton effect describes magnetic-field-induced birefringence in liquids. It is the magnetic analog of the Kerr effect, in which an applied electric field induces birefringence. Additionally, the Cotton–Mouton effect can be viewed as the liquid-phase counterpart to the Voigt effect, which occurs in gaseous media. The magnetic field causes a partial alignment of the molecular or structural anisotropies within the medium, leading to a directional dependence of the refractive index. This effect is quadratic with respect to the magnetic field strength and is conceptually analogous to the Kerr effect, which involves the induction of birefringence by an external electric field—thus categorized as an electro-optical effect. The Cotton–Mouton effect occurs at the molecular level when a transverse magnetic field disrupts electron motion in molecules, causing minor differences in their effective polarizabilities. In liquids, where molecules typically have random orientations, this disruption leads to a slight alignment of molecular or structural anisotropies. As a result, the refractive index varies with direction, resulting in birefringence.

In contrast to the Faraday effect, which also arises due to the interaction between light and a magnetic field, the Cotton–Mouton effect does not involve a rotation of the polarization plane but rather a differential phase shift between orthogonal polarization components. While the Faraday effect is linear in the magnetic field and depends on the direction of light propagation relative to the field, the Cotton–Mouton effect is quadratic and occurs only when the magnetic field is transverse to the light beam. Together, these effects are central to the study of magneto-optical properties of materials and play an important role in precision measurements, nonlinear optics, and the characterization of magnetic fluids and plasmas.

In recent years, the spotlight on magneto-optical and electro-optical effects in transparent ceramics has grown quite dramatically, thanks to improvements both in material preparation and optical measurement tools. Transparent ceramics—whether bulk (single-crystal [3] or polycrystalline [4,5,6]) or as thin films—offer a rare combination: optical transparency, chemical and thermal robustness, and a tuneable structure that responds to magnetic or electric fields. They can host strong Kerr, Faraday or Cotton–Mouton responses, and so they are gaining traction for devices like optical isolators, modulators, or sensors. On the materials side, sophisticated fabrication routes such as sol-gel [7,8] and hydrothermal wet chemistry [9], conventional solid-state routes [10], and densification methods like spark plasma sintering (SPS) [11,12,13], high-pressure/high-temperature processing, controlled melting [3], or hot isostatic pressing [14] have made bulk ceramics far more accessible. At the same time, thin-film approaches such as chemical vapor deposition (CVD) [15], physical vapor deposition (PVD) methods, like sputtering [16], and radio frequency magnetron sputtering [17,18,19] allow for precise compositional and microstructure control. For thicker layers, spin coating is often employed [20].

The intersection of optics and magnetism in contemporary materials science necessitates a concentrated review that consolidates the existing knowledge of Kerr-type effects in transparent ceramics and elucidates their fundamental mechanisms. This review is crucial from a molecular perspective, as Kerr-type phenomena stem from field-induced disruptions in the electronic structure and polarizability of the ions and molecular units in the ceramic lattice. Grasping how these microscopic interactions culminate in macroscopic magneto-optical responses is vital for enhancing the design and optimization of future magneto-optical materials.

## 2. Molecular Mechanisms of Magneto- and Electro-Optical Effects

The next section offers an in-depth explanation of the intricate physical mechanisms that underpin the Kerr, Faraday, and Cotton–Mouton effects. In this discussion, we will highlight the crucial molecular interactions that play a significant role in these phenomena. These interactions include, but are not limited to, field-induced alterations in electronic structure, variations in polarizability, and the alignment of molecules in response to external fields. This approach establishes a solid foundation for the subsequent discussion of material properties and behavior.

### 2.1. Magneto-Optical Faraday Effect

A key distinguishing feature of the magnetooptical Faraday effect is that the rotation direction is invariant with respect to the direction of light propagation relative to the magnetic field—i.e., whether the light travels in the same or the opposite direction as B, the observed rotation is always in the same sense when viewed along the field direction. This contrasts with natural optical activity observed in optically active materials, where chiral media exhibit either right- or left-handed rotation depending on the substance’s handedness.

The rotation angle θ (in radians) of the polarization plane is proportional to the optical path length l (in cm) through the material and the magnetic field strength B (in Gauss), as described by the following empirical relation (Equation (1)) [21]:

(1) θ=V·B·l

here, V is the Verdet constant. The Verdet constant has units of $\mathrm{rad}\cdot G^{-1}\cdot {\mathrm{cm}}^{-1}$ [22].

From the standpoint of molecular physics, the Faraday effect arises from the Zeeman splitting of atomic or molecular energy levels in the presence of a magnetic field. This field-induced splitting of fine-structure or rotational-vibrational levels leads to magnetically induced optical anisotropy (a form of induced birefringence), which is responsible for the observed rotation. Fundamentally, the interaction between the light wave and the magnetic field is mediated through the electronic and, in molecules, also the vibronic states of the atoms or molecular units in the medium.

Since Zeeman sublevels are associated with specific polarization states, the incident linearly polarized light—composed of a superposition of left- and right-circularly polarized components—interacts differently with each sublevel, depending on the direction of angular momentum precession [23]. In molecular systems, additional contributions can arise from the field-induced coupling between rotational, vibrational, and electronic motions. The light’s interaction with the sample thus depends on the polarization type and its alignment with respect to the magnetic field B which serves as the quantization axis:

- linear polarization E ‖ B corresponds to π -polarized transitions, satisfying the selection rule Δm=0,
- linear polarization E ⊥ B corresponds to σ-polarized light, which is a superposition of left (−σ) and right (+σ) polarized components, with selection rules Δm=±1,
- right-circular polarization (+σ): Δm=+1,
- left-circular polarization (−σ): Δm=−1.

A linearly polarized wave can be decomposed into equal-amplitude left and right-circular components, which rotate in opposite directions. As this wave propagates through a medium of refractive index *n* and length *l*, the phase difference between the two circular components increases, leading to a rotation of the recombined polarization plane. The phase shift θ is given by the Equation (2):

(2) θ=n·k·l

where the wavevector *k* is defined as (Equation (3)):

(3) $$k=\frac{2\pi }{\lambda }$$

where λ is wavelength of light (in nm). Since the left- and right- circular components experience different refractive indices due to the Zeeman effect, they accumulate different phase shifts during propagation. Upon recombination, this results in a net rotation of the polarization plane. Complementary to this, circularly polarized light can generate a non-equilibrium magnetization in otherwise nonmagnetic materials through the inverse Faraday effect (IFE) [24,25]. Initial theoretical frameworks for this phenomenon were developed specifically for nonabsorbing media. In molecular systems, the coupling between electronic, vibrational, and rotational degrees of freedom further modulates this effect, making the induced magnetization strongly dependent on molecular structure, orientation, and the polarization of the incident light. In general, the magnetization associated with IFE arises from the light-induced orbital motion of charges or from the photoexcitation of electrons into spin-polarized states [26,27,28]. Together, the Faraday and inverse Faraday effects illustrate how magnetic fields and light interact at both the atomic and molecular levels, highlighting the importance of electronic and vibronic structure in determining magneto-optical responses in materials.

### 2.2. Kerr Effects

When an initially isotropic medium (i.e., one with uniform optical properties in all directions) is subjected to an external electric field, the induced change in the refractive index can vary depending on the polarization direction of the incident light relative to the field orientation. At the molecular level, this arises because the applied field perturbs the electronic clouds of the constituent molecules, inducing anisotropic polarizability. This direction-dependent molecular response leads to field-induced birefringence, resulting in differing refractive indices for orthogonal polarizations. Due to the finite spectral bandwidth of femtosecond laser pulses, and the fact that the refractive index is frequency-dependent (a consequence of molecular resonances that give rise to dispersion), the temporal profile of the pulse broadens during propagation through a dispersive medium. This pulse-stretching effect becomes more significant as the pulse duration decreases. The intensity-dependent refractive index change induced by the propagating pulse itself, originating from the nonlinear polarization of molecules, can be harnessed when appropriately shaped to compensate for dispersion and effectively counteract pulse broadening. This has promising implications for high-speed optical fiber communication, where minimizing pulse duration allows for reduced time intervals between consecutive pulses, enabling faster data transmission over long distances.

The optical Kerr effect also plays a significant role in laser spectroscopy. One important application is the creation of ultrafast, optically controlled shutters [29,30]. For instance, in femtosecond fluorescence measurements, one laser pulse excites the sample, while a second, time-delayed pulse activates the Kerr shutter. This allows detection of fluorescence emission only after a precise delay τ, with temporal resolution determined by the duration of the control pulse. Beyond its use in shutter systems, the optical Kerr effect serves as an independent spectroscopic technique.

In time-resolved spectroscopy, Kerr-based pump–probe measurements provide insight into ultrafast molecular dynamics by tracking the relaxation of field-induced anisotropy. This response reveals rotational diffusion, vibrational dephasing, and intermolecular structural rearrangements on femtosecond to picosecond timescales. In many materials, the Kerr-induced birefringence does not vanish instantaneously after the laser pulse but instead decays over time scales ranging from femtoseconds to tens of picoseconds, and in some cases up to nanoseconds. This decay reflects the molecular vibrations, rotations, and intermolecular structural dynamics within the medium. By analyzing the time evolution of the Kerr signal, one can extract depolarized Raman spectra [31]. A key advantage of this technique is its ability to detect low-frequency vibrational modes, since the Rayleigh line is absent from the observed spectrum. Moreover, the ability to analyze the signal in the time domain allows for precise measurements of coherence times associated with oscillatory modes, enhancing the temporal resolution of spectroscopic studies [32,33]. The Kerr effect is also used in optical pulse characterization techniques, such as self-referencing methods for measuring ultrashort pulse widths via Kerr-induced self-diffraction or intensity-dependent phase modulation [34]. In nonlinear photonics, Kerr media serve as the basis for all-optical switching, logic gates, and mode-locking mechanisms in ultrafast lasers, where the intensity-dependent refractive index enables fast, electronic-free modulation of optical signals [35,36].

Later, it found practical applications in devices such as Kerr shutters, which are used in high-speed photography. The switching time of such shutters can be as short as 1 nanosecond. Nearly a century later, the optical Kerr effect is observed following the advent of high-intensity laser sources. In this regime, the propagating laser beam itself modifies the refractive index of the medium in which it travels. When the refractive index increases with light intensity, the central part of the beam—where intensity is highest—travels more slowly than the edges. This causes the wavefront (i.e., the surface of constant phase) to bend, leading to a phenomenon known as self-focusing, as the beam effectively acts as its own lens [30,37].

In the present work, a brief overview of theoretical and experimental approaches to the Kerr effect reported in the literature is provided. According to early statistical–mechanical studies, a general framework was developed to describe the dielectric properties of fluid systems, forming a foundation for molecular-level analyses of nonlinear optical effects such as the Kerr effect [38]. According to the authors of Ref. [39], a compact theoretical expression for the Kerr constant of a low-pressure gas was derived from the general form of the molecular energy in a strong electric field. It was shown that the temperature-independent contribution to the Kerr constant is directly related to the field dependence of the molecular polarizability, and that for spherically symmetric molecules, the Kerr constant provides direct access to the corresponding hyperpolarizability. In another approach, a time-dependent variational formulation based on double perturbation theory was presented for the hyperpolarizabilities relevant to the Kerr effect [40]. As demonstrated in that work, the method enables a quantitative description of Kerr phenomena and was successfully applied to the hydrogen atom, yielding accurate Kerr dispersion curves over a wide spectral range, including the first two lines of the Lyman series. According to recent studies of molecular dynamics in the terahertz regime, intermolecular hydrogen-bond vibrations, molecular reorientation processes, and solute–hydrogen-bond interactions occur predominantly in the THz frequency range and are closely linked to the physical, chemical, and structural dynamics of molecular systems [41]. As emphasized in that work, the identification of individual modes remains challenging due to their collective and cooperative nature and the strong spectral overlap of the associated excitations. More recently, Kerr phenomena have also been investigated in the context of topological and magnetic materials. In Ref. [42], the authors introduced a general-frequency theory of axion electrodynamics and defined an optical axion magnetoelectric coupling through its relation to the optical surface Hall conductivity. According to that study, axion electrodynamics can give rise to a pronounced Kerr effect in thin-film antiferromagnets, even at wavelengths much larger than the characteristic length scale of magnetic modulation. Finally, as shown in Ref. [43], optical methods constitute powerful probes of the electronic and spin structure of materials. Using a tight-binding model combined with linear response theory, the authors investigated the magneto-optical Kerr and Faraday effects in two-dimensional second-order topological insulators subjected to external magnetization, further extending the applicability of Kerr spectroscopy to emerging quantum materials.

#### 2.2.1. Mathematical Principles of Magneto-Optical Kerr Effect

**Figure 1:**
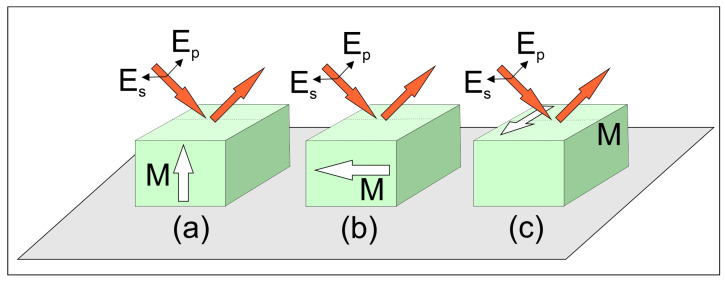
Three types of experimental geometry for MOKE measurement: (**a**) polar Kerr effect, (**b**) longitudinal Kerr effect, and (**c**) transverse Kerr effect. E_*p*_—parallel polarization and E_*s*_—perpendicular polarization—two fundamental orthogonal linear polarization states of light.

The MO Kerr effect can be studied under broadly applicable conditions, allowing the magnetization direction of the sample to be oriented arbitrarily with respect to the plane in which the incident light propagates. At the microscopic level, the interaction arises because the magnetic field couples to the electronic and spin degrees of freedom of the material: the magnetization modifies the complex dielectric tensor through spin–orbit coupling and field-induced anisotropy in the molecular or atomic electronic transitions. Nonetheless, three specific configurations (Figure 1) are particularly significant and will be examined individually in this article. When the magnetization vector M lies within the sample surface and aligns with the plane of incidence, the effect is referred to as the longitudinal Kerr effect. If M is oriented perpendicular to the surface of the sample, the resulting effect is termed the polar Kerr effect. In contrast, when M is parallel to the surface but oriented perpendicular to the plane of incidence, the phenomenon is known as the transverse Kerr effect. The underlying physical principle of the Kerr effect is fundamentally the same as that of the Faraday effect; both can be described using the same theoretical framework. The key difference lies in their manifestation—the Kerr effect occurs in reflection, whereas the Faraday effect is observed in transmission. Both phenomena originate from magnetically induced circular birefringence and dichroism, caused by Zeeman splitting of energy levels and the resulting modification of transition dipoles and selection rules.

In the polar configuration of the Kerr effect, the magneto-optical response becomes more pronounced as the angle of incidence approaches normal. The effect reaches its maximum at normal incidence, whereas in the longitudinal and transverse geometries, it diminishes and effectively vanishes under the same conditions. Consequently, when comparing all three standard Kerr geometries across varying angles of incidence, the polar configuration at normal incidence yields the strongest magneto-optical signal, making it particularly advantageous for applications such as magneto-optical data storage. This behavior arises from the distinct way in which the magnetization couples to the optical field in each geometry. In the polar configuration, the magnetization is perpendicular to the sample surface, and thus to the plane of incidence, allowing the induced circular birefringence and dichroism—originating from magnetically split electronic transitions—to directly influence both s- and p-polarized components of the reflected light. As a result, the off-diagonal elements of the dielectric tensor contribute maximally to the Kerr rotation and ellipticity when the incidence is normal.

In contrast, in the longitudinal and transverse geometries, the magnetization lies within the plane of the surface. Under normal incidence, the electric field of the incoming light lacks the necessary component parallel to M to induce a significant magneto-optical coupling. From a microscopic perspective, the projection of the optical field onto the magnetization determines the extent to which spin–orbit-coupled electronic states experience differential excitation. When this projection goes to zero at normal incidence, the induced anisotropy in the refractive index—and hence the Kerr response—collapses [44,45]. Consequently, while the polar configuration remains robust even at normal incidence, the longitudinal and transverse effects inherently rely on oblique incidence to generate measurable changes in polarization.

The electromagnetic basis of the magneto-optical Kerr effect can be described using the dielectric tensor ε, which characterizes how the medium responds to the electric field of light in the presence of a magnetic field or internal magnetization. This response arises from the interaction of the light’s electric field with the electrons in atoms or ions, whose energy levels and transition dipoles are influenced by the local magnetic moments.

For a medium magnetized along the Z-axis, the dielectric tensor takes the following form:

(4) $$\varepsilon =\left(\begin{array}{ccc} \varepsilon_{xx} & i\varepsilon_{xy} & 0\\ -i\varepsilon_{xy} & \varepsilon_{yy} & 0\\ 0 & 0 & \varepsilon_{zz} \end{array}\right)$$

In the case of an isotropic material, the diagonal elements are equal:

(5) $$\varepsilon_{xx}=\varepsilon_{yy}=\varepsilon_{zz}=\varepsilon$$

and the off-diagonal term is purely imaginary and antisymmetric:

(6) $$\varepsilon_{xy}=-\varepsilon_{yx}$$

thus, the dielectric tensor simplifies to:

(7) $$\varepsilon =\left(\begin{array}{ccc} \varepsilon & i\varepsilon^{\prime } & 0\\ -i\varepsilon^{\prime } & \varepsilon & 0\\ 0 & 0 & \varepsilon \end{array}\right)$$

where:

- ε is the base (possibly complex) dielectric constant of the material, arising from the collective electronic polarizability of the molecules,
- $\varepsilon^{\prime }$ represents the magneto-optical contribution, which is generally small and may be complex in absorbing materials. Microscopically, $\varepsilon^{\prime }$ reflects the spin–orbit coupling of electrons and their Zeeman splitting in the internal magnetic field, leading to a difference in response to left- and right-circularly polarized light.

The complex refractive indices can be expressed as:

(8) $$N_{\pm }=\varepsilon_{xx}\pm i\varepsilon_{xy}$$

where:

(9) $$N_{+}=n_{+}+i\kappa_{+},\;N_{-}=n_{-}+i\kappa_{-}$$

here, n_+_ and n_−_ denote the refractive indices, and $\kappa_{+}$ and $\kappa_{-}$ are the extinction coefficients for right- and left-handed circularly polarized light, respectively, while *i* is the imaginary unit.

Since the difference between N_+_ and N_−_ is typically small, we define:

(10) $$\Delta n=n_{+}-n_{-},\;\Delta \kappa =\kappa_{+}-\kappa_{-},\;n=\frac{n_{+}+n_{-}}{2},\;\kappa =\frac{\kappa_{+}+\kappa_{-}}{2}$$

and decompose the components of the dielectric tensor as:

(11) $$\varepsilon_{xx}=\varepsilon_{xx}^{\prime }+i\varepsilon_{xx}^{\prime \prime },\;\varepsilon_{xy}=\varepsilon_{xy}^{\prime }+i\varepsilon_{xy}^{\prime \prime }$$

With these definitions, the differences in refractive index and extinction coefficient are given by:

(12) $$\Delta n=\frac{\kappa \varepsilon_{xy}^{\prime }-n\varepsilon_{xy}^{\prime \prime }}{n^{2}+\kappa^{2}}$$

(13) $$\Delta \kappa =\frac{n\varepsilon_{xy}^{\prime }+\kappa \varepsilon_{xy}^{\prime \prime }}{n^{2}+\kappa^{2}}$$

When a polarized light beam propagates through a medium in the direction of an applied magnetic field B, the medium responds differently to the right- and left-circularly polarized (RCP and LCP) components of the light. This difference arises because the RCP and LCP components drive electronic transitions that are selectively shifted in energy by the magnetic field due to spin–orbit and exchange interactions. This results in distinct refractive indices $n_{\pm }=\sqrt{\varepsilon \pm i\gamma }$. Since Fresnel reflection coefficients are functions of the refractive index, the RCP and LCP components are reflected with different amplitudes, $r_{+}$ and $r_{-}$. If a phase difference exists between these components, the reflected light undergoes a rotation in its polarization state. Additionally, if the magnitudes $|r_{+}|$ and $|r_{-}|$ differ, the reflected light becomes elliptically polarized. All three Kerr effects can be generally described using a tensor of reflection coefficients:

(14) $$r=\left(\begin{array}{cc} r_{ss} & r_{sp}\\ r_{ps} & r_{pp} \end{array}\right)$$

The elements of the matrix represent the reflection and cross-polarization conversion coefficients. For example, r_*sp*_ describes the reflection of a p-polarized incident wave into an s-polarized reflected wave. The superscripts, which are often omitted in matrix notation, indicate the incident and reflected field components.

In the special case of a transparent medium where n_±_ are real, there is no phase shift between r_+_ and r_−_; however, unequal magnitudes can still induce ellipticity in the reflected beam. Conversely, for an absorbing medium—where both ε and γ are complex—the reflected light typically exhibits elliptical polarization with the ellipse’s major axis rotated relative to the polarization direction of the incident light. The complex Kerr effect can be characterized by the parameter γ, defined as:

(15) $$\gamma =\frac{r^{+}-r^{-}}{r^{+}+r^{-}}$$

where r^+^ and r^−^ are the complex reflection coefficients for right- and left-circularly polarized light, respectively. These coefficients can be expressed in terms of the linear polarization basis (s and p) as follows:

(16) $$r^{\pm }=\frac{1}{2}\left(r_{ss}+r_{pp}\pm i\left(r_{sp}-r_{ps}\right)\right)$$

As a result, the reflected light becomes elliptically polarized, but the major axis of the polarization ellipse remains aligned with the polarization direction of the incident light—i.e., there is ellipticity but no Kerr rotation.

Conversely, in an absorbing medium—where both the dielectric function ε and the Kerr parameter γ are complex—the reflected light exhibits elliptical polarization with both ellipticity and rotation of the polarization ellipse relative to the incident polarization. In this case, γ is complex and can be written as:

(17) $$\gamma =\gamma e^{i\phi }$$

where the real and imaginary parts correspond to the Kerr rotation angle $\theta_{K}$ and ellipticity $\varepsilon_{K}$, respectively:

(18) $$\theta_{K}+i\varepsilon_{K}\propto \gamma$$

#### 2.2.2. Primary and Secondary Magneto Optic Kerr Effects

Within the framework of the MOKE, the response can be further categorized into primary and secondary Kerr effects. The primary Kerr effect refers to the direct, typically linear, interaction between the magnetization and the optical field, which leads to measurable changes in the polarization state of reflected light. This effect is often dominant in materials with high crystal symmetry and strong spin-orbit coupling, where the magnetization induces a straightforward modification of the dielectric tensor. It can be accurately described by a first-order magneto-optical tensor and is responsible for the characteristic Kerr rotation and ellipticity commonly observed in ferromagnetic thin films [46]. Furthermore, the primary Kerr effect provides a sensitive probe of the magnetization direction and magnitude, enabling applications such as magnetic domain imaging and magnetization dynamics studies [47]. In addition to these macroscopic mechanisms, the primary Kerr response is inherently linked to molecular- and atomic-scale properties, particularly those governing the electronic structure. The strength and symmetry of the magneto-optical tensor depend on the distribution of electronic orbitals, the degree of orbital hybridization, and the local crystal fields acting on magnetic ions. Spin–orbit coupling, which mediates the interaction between the spin polarization and the optical field, originates from relativistic corrections to the electronic motion within individual atoms and molecular units. As a result, materials with heavy elements, strong ligand fields, or highly anisotropic coordination geometries exhibit enhanced Kerr signals. Moreover, molecular-level factors such as charge-transfer transitions, the density of states near the Fermi level, and exchange splitting between spin-polarized bands can substantially modify the frequency dependence and magnitude of the observed Kerr rotation. These microscopic contributions are especially relevant in systems with organic–inorganic interfaces, molecular magnets, or low-dimensional heterostructures, where local bonding and orbital symmetry strongly influence the magneto-optical response.

On the other hand, the secondary Kerr effect involves more subtle and complex contributions to the magneto-optical response. These may arise from structural anisotropies, strain-induced modifications, higher-order terms in magnetization (quadratic or nonlinear magneto-optical effects), or mixed tensor components that couple different physical mechanisms [48,49]. Such secondary effects often manifest as changes in the intensity or polarization state of the reflected light that do not scale linearly with magnetization, complicating the interpretation of magneto-optical measurements [50]. Additionally, secondary Kerr effects become significant in low-symmetry materials or under conditions where multiple scattering and interface effects play a role, thereby providing richer information about the material’s magnetic and structural properties [51]. At the microscopic level, these secondary contributions may also reflect molecular-scale distortions, such as variations in bond lengths, octahedral tilting, Jahn–Teller effects, or dynamic lattice fluctuations that perturb the local electronic environment. Such perturbations modify the higher-order terms of the dielectric tensor and can couple nonlinearly to magnetization, giving rise to anisotropic or frequency-dependent secondary Kerr signatures that encode detailed information about local bonding and symmetry breaking [52,53].

Table 1 summarizes the various orders of the Kerr effect.

#### 2.2.3. Mathematical Principles of Electro-Optical Kerr Effect

**Figure 2:**
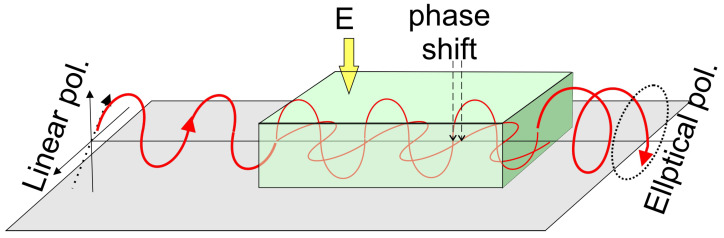
The mechanism of the magneto-optic Kerr effect. Linearly polarized light passes through a region with an applied electric field. As a result, the light emerges with elliptical polarization, signifying a change in the medium’s refractive index. Dotted arrows indicate polarization plane.

Kerr discovered that when an isotropic transparent material is subjected to an electric field, it exhibits birefringence. The medium behaves like a uniaxial crystal, with its optical axis aligning with the direction of the applied electric field, as illustrated in the Figure 2.

The influence of an electric field on the optical properties of a crystal can be considered in terms of changes in the refractive index ellipsoid, which corresponds to variations in the dielectric impermeability tensor $\eta_{ij}$.

For small changes in the shape, size, and orientation of the refractive index ellipsoid, these variations can be described by the Equation (19):

(19) $$\Delta \eta_{ij}=\eta_{ij}^{0}-\eta_{ij}$$

Here, $\eta_{ij}^{0}$ denotes the dielectric impermeability tensor of the undeformed crystal, while $\eta_{ij}$ refers to the tensor of the deformed crystal.

In the case of small perturbations (for small electric fields) of the tensor $\eta_{ij}^{0}$, its dependence on the components of the electric field vector E_*k*_ can, with a good approximation (in the linear approximation), be expressed as (Equation (20)):

(20) $$\Delta \eta_{ij}=r_{ijk}E_{k}$$

which form r_*ijk*_ are the components of a third-rank tensor, are called electro-optic coefficients. The linear electro-optic effect described by the Equation (21) is commonly referred to as the Pockels effect. In matrix form, the Pockels effect can be written as:

(21) $$\Delta \eta_{m}=r_{mk}\cdot E_{k}$$

where:

- r_*mk*_ are the electro-optic (Pockels) coefficients. They form a 6×3 matrix obtained by contracting the third-rank tensor r_*ijk*_, which relates the change of the dielectric impermeability tensor to the applied electric field.
- E_*k*_ are the components of the external electric field vector, where k = 1, 2, 3 correspond to the Cartesian components E_*x*_, E_*y*_, E_*z*_.

Thus, the product r_*mk*_ E_*k*_ describes how the electric field modifies the optical properties of the crystal via the linear electro-optic (Pockels) effect.

The electro-optic tensor r_*ijk*_ is a material tensor; and as such, it is subject to symmetry restrictions imposed by Neumann’s principle. Since the electro-optic tensor is of third rank, the linear electro-optic effect does not occur in crystals that possess a center of symmetry.

Two types of linear electro-optic effects can be distinguished:

- When the crystal cannot deform, the electro-optic effect observed in such a constrained crystal is called the primary electro-optic effect.
- In contrast, in a free crystal, the electric field induces mechanical deformations due to the inverse piezoelectric effect. These deformations lead to changes in the refractive indices via the elasto-optic effect. The electro-optic effect observed in this case is referred to as the secondary electro-optic effect.

In a free (unconstrained) crystal, the total electro-optic effect is thus the sum of the primary and secondary effects, and the resulting change in the dielectric impermeability tensor is given by the Equation (22):

(22) $$\Delta \eta_{ij}=r_{ijk}\cdot E_{k}+p_{ijkl}\cdot d_{kml}E_{m}=\left(r_{ijk}+p_{ijmn}d_{nmk}\right)E_{k}$$

here, d_*nmk*_ are the piezoelectric coefficients, and p_*ijmn*_ are elasto-optic coefficients.

The linear electro-optic effect is typically observed under conditions where the electric field-induced stresses and crystal deformations are small.

For large electric fields and significant deformations, it becomes necessary to include quadratic terms in Equation (12), leading to the Equation (23):

(23) $$\Delta \eta_{ij}=r_{ijk}\cdot E_{k}+R_{ijkl}\cdot E_{k}E_{l}$$

The EOKE can strongly reorient anisotropic molecules when subjected to intense electric or optical fields. In liquids containing elongated or highly polarizable species, such fields tend to align molecular axes with the polarization direction, generating birefringence that cannot be explained by electronic polarization alone. When this field-driven torque becomes comparable to intermolecular forces such as steric interactions or dipole–dipole coupling—the molecular ensemble may reorganize cooperatively, shifting from an almost isotropic arrangement toward a more ordered, mesophase-like state reminiscent of nematic liquid crystals. This interplay between external fields and molecular interactions amplifies the nonlinear optical response and, under suitable conditions, can even produce field-induced first-order phase transitions, as shown in foundational studies of anisotropic molecular fluids [54].

#### 2.2.4. Primary and Secondary EO Kerr Effects

Like the Pockels effect, the Kerr effect can be primary or secondary:

- Primary Kerr effect occurs in non-deformable (clamped) crystals.
- Secondary Kerr effect arises in deformable (free) crystals, where electrostriction and the inverse piezoelectric effect lead to strain-induced birefringence.

This is described by the Relation (24):

(24) $$r_{jk}=d_{ijk}\cdot E_{i}+\gamma_{imjk}E_{i}\cdot E_{m}$$

here, the first term in the Equation (24) corresponds to the linear piezoelectric effect, while the second quadratic term represents electrostriction. The deformations caused by electrostriction lead to changes in the refractive indices via the elasto-optic effect.

The total change in dielectric impermeability in a free crystal is then $\eta_{ij}$, given by Equation (25):

(25) $$\Delta \eta_{ij}=R_{ijkl}E_{k}E_{l}+p_{ijmn}\gamma_{mnkl}E_{k}E_{l}=\left(R_{ijkl}+p_{ijmn}\gamma_{mnkl}\right)E_{k}E_{l}$$

here, $\gamma_{mnkl}$ are electrostrictive coefficients.

In crystals possessing a center of inversion symmetry, the linear electro-optic effect (Pockels effect) is absent due to symmetry constraints. Therefore, the dominant electro-optic phenomenon in such materials is the Kerr effect.

#### 2.2.5. Nonlinear Optical Response

**Figure 3:**
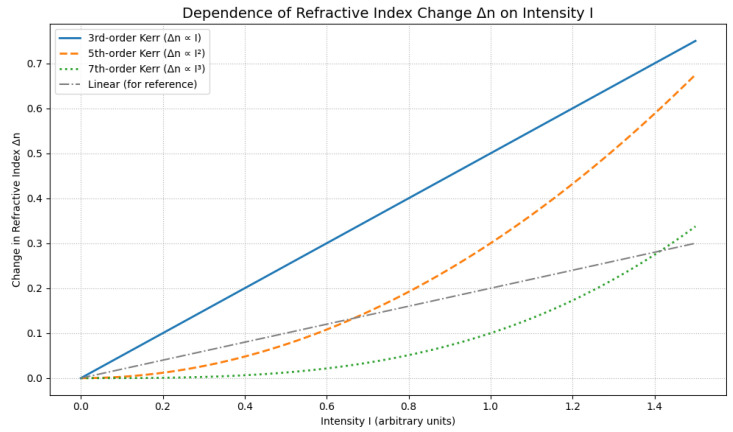
The dependence of the refractive index on light intensity for different orders of the Kerr effect.

Figure 3 illustrates the dependence of the refractive index on light intensity for different orders of the Kerr effect. The plot highlights how higher-order nonlinearities progressively modify the refractive index with increasing light intensity, providing insight into the transition from the linear regime to strong-field optical responses. Table 1 shows the various orders of the electro-optic Kerr effect.

## 3. Measurement Methods of Kerr Effects

For studies of the Faraday, Kerr, and Cotton–Mouton effects, similar measurement setups, experimental schemes, optical components, and detection systems can be employed. The main differences arise from the specific configuration of the optical arrangement and the methods used to analyze the collected data. In this paragraph, we focus primarily on describing the design and operating principles of typical measurement systems used for Kerr-effect studies. For clarity, these systems are grouped into several categories, ranging from the simplest arrangements to those employing phase modulation, interferometry, microscopy, and synchrotron-based techniques. These experimental setups enable the investigation of phenomena occurring at the atomic scale, the molecular scale, and up to macroscopic dimensions. The use of high-sensitivity, high-precision instrumentation allows increasingly accurate characterization of changes induced in various media by external stimuli. This aspect is often overlooked in the literature; therefore, we aim to present the most important solutions and measurement approaches.

### 3.1. Experimental Setups for Magneto-Optical Kerr Effect

**Figure 4:**
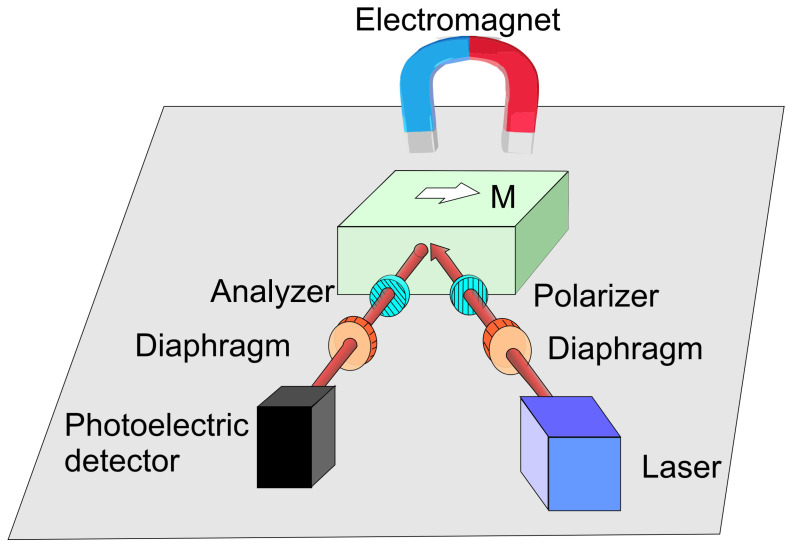
Basic components of the experimental setup of MOKE. A white arrow shows the direction of the magnetic field. Electromagnet color represent blue: north pole and red: south pole.

A critical aspect of magneto-optical Kerr effect measurements, as with many optical techniques, lies in the precise illumination of the sample and efficient coupling of reflected light into a photodetector. While the fundamental MOKE setup can be remarkably simple, as illustrated in Figure 4, precise alignment remains essential for accurate results.

In this basic configuration, monochromatic light passes through a polarizer (P) and is reflected off a mirror (or later, a magneto-optical sample) before reaching a detector through an analyzer (A). With the polarizer fixed, the analyzer is rotated to minimize the detector signal—establishing a reference condition in the absence of magnetic influence. Replacing the mirror with a magneto-optical sample and applying a magnetic field leads to a deviation from this minimum. The analyzer must then be rotated by an angle θ, the Kerr rotation, to restore minimal signal. However, $\theta_{K}$ is typically very small, as John Kerr remarked, “the effect is very faint at best” [55].

Historically, Nicol prisms were used for generating and analyzing linearly polarized light. Their advantage lies in producing fully polarized beams without altering propagation direction. However, they have largely been replaced by more efficient polarizing elements such as Glan–Thompson, Glan–Foucault, Rochon, and Wollaston prisms. When aligned correctly, the extraordinary beam from the first Nicol prism can be completely extinguished by the second. Upon applying a magnetic field, complete extinction is no longer possible—this residual transmission is a signature of the Kerr effect. This process is fully reversible, as the signal reverts to extinction when the magnetic field is removed. Furthermore, accurate polarization analysis often requires compensating for wavelength-dependent phase shifts, typically addressed using retarding plates. This becomes particularly problematic for measurements involving wavelength scans, as a different retarder is required for each wavelength, hindering automation.

The basic experimental setup was presented in Ref. [56]. The incident laser beam is first directed through a polarizer and a diffraction grating before interacting with the sample subjected to an external magnetic field. The light reflected from the sample subsequently passes through a second polarizer (acting as an analyzer) and a convex lens before being detected by a photodetector. In the absence of a magnetic field, the analyzer is initially oriented to achieve complete extinction of the signal. When a magnetic field is applied, the polarization plane of the reflected light experiences a field-dependent rotation, leading to a measurable variation in the detected signal intensity. This variation enables the establishment of a quantitative relationship between the applied magnetic field and the corresponding reflected light intensity from the sample. The process begins with the laser passing through the polarizer, producing linearly polarized light. This light is reflected by the sample in the presence of a magnetic field and then passes through the analyzer before reaching the photodetector. Simultaneously, a magnetometer monitors the magnetic field strength and converts it into an electrical signal. Both the magneto-optical signal and the magnetic field data are acquired and visualized using dedicated software on a computer, enabling comprehensive analysis of the sample’s magneto-optical behavior.

To study how magnetization behaves in materials when a magnetic field is applied perpendicular to the surface, researchers typically use laser-based polar MOKE techniques [57,58,59,60,61,62,63,64,65,66,67,68] or by applying (anomalous) Hall effect transport measurements [64,69,70,71,72].

Significant research has focused on improving the spatial resolution and signal-to-noise ratio in MOKE measurements. Common, these advancements have been pursued using static (DC) or oscillating (AC) magnetic fields generated by bulky electromagnets driven by high-current power supplies, typically achieving field strengths in the range of 1–2 T [44,73,74]. A more advanced and efficient methodology involves modulating the laser probe in the presence of a time-dependent magnetic field, with the magneto-optical signal extracted using phase-sensitive detection techniques such as lock-in amplification. Pulse-field MOKE measurements are particularly demanding, as the Kerr-induced polarization rotations are typically on the order of milliradians. The small changes in light intensity, which must be monitored by the detector, are well-suited for precise measurement using lock-in amplifiers [75,76,77,78]. As a result, successful implementations remain rare, with most studies conducted at room temperature and magnetic fields between 10 T [79,80] and 40 T. Extending such techniques to more extreme regimes requires both highly sensitive detection methods and mechanically stable, alignment-free optical architectures. Both commercial and custom-built spectrometers have been employed for such applications. A key instrumental component typically added is a white light source combined with a monochromator. Foundational work from the Schoenes group provides detailed descriptions of the spectrometer setups used in similar studies [81].

An advanced optical measurement system, which present technique provides a sensitivity of 0.002° for Kerr rotation at 500 nm was presented by Sato [82]. A broadband light beam was generated using a 150 W halogen-tungsten lamp, which necessitated the use of a monochromator to narrow the spectral bandwidth of the incident light. A mirror assembly directed the beam through a polarizer and focused it onto the surface of the sample. A key innovation in this setup was the implementation of a piezo-birefringent modulator, which enabled the generation of a periodically varying birefringence within the optical path. This, in turn, resulted in a time-dependent modulation of the light intensity. Additionally, the optical beam was mechanically interrupted prior to polarization using a chopper.

After reflection from the sample surface, the light passed through a crystalline analyzer and was subsequently detected by either a photodetector, a photomultiplier tube (PMT), or an indium antimonide (InSb) photocell. Due to the use of modulation techniques, lock-in amplifiers were employed to extract and amplify the measurement signals. Notably, three lock-in amplifiers were used in this setup: one dedicated to analyzing the polarization state, and two others to measure parameters related to the beam intensity. The output signal was acquired and further processed using dedicated computer software, enabling precise and sensitive optical measurements.

While traditional MOKE systems used free-space optics—with precise lens and mirror alignments to guide the beam—these setups face significant limitations under extreme conditions, such as high magnetic fields or cryogenic temperature [83]. To overcome these challenges, authors propose using a fiber-based, loop-less Sagnac interferometer for pulse-field MOKE measurements [84,85,86,87]. An example of the measurement setup is presented in Figure 4. This approach eliminates alignment complexities and enhances system robustness under non-ambient conditions. In a Sagnac interferometer, a light beam is split into two components that propagate in opposite directions along the same optical path. This counter-propagation is induced by reflection at the sample. Upon returning to the entry point, the beams exit the loop, and a polarization-maintaining (PM) circulator redirects the light toward the detector. High-precision measurements of the polar Kerr effect in the spin-triplet superconductor Sr_2_RuO_4_ were conducted using a zero-area Sagnac interferometer [88]. The light source is a superluminescent diode (SLED) with a very short coherence length and a central wavelength of 1550 nm. Its output is directed through a circulator and polarizer. A half-wave (λ/2) plate then rotates the polarization by 45° relative to the axes of a birefringent electro-optic modulator (EOM), which operates at 5.078 MHz—twice the optical transit frequency of the system. The EOM splits the beam into two orthogonally polarized components, which are coupled into the fast and slow axes of fiber, which is routed into a ^3^He cryostat (T < 0.5 K). An aspheric lens focuses the emerging light through a -quarter-wave plate (QWP, λ/4) onto the surface of a Sr_2_RuO_4_ sample. The λ/4 plate, aligned at 45° to the PM fiber axis, converts the orthogonally polarized beams into left- and right-circularly polarized light. Upon reflection from the TRS-breaking sample, the two circular polarizations acquire a nonreciprocal phase shift. $\Delta \phi_{\mathrm{nr}}=2\theta_{K}$, where $\theta_{K}$ is the Kerr rotation. The QWP converts the reflected beams back into linear polarizations, with a net 90° rotation of their polarization axes, effectively causing the beams to exchange optical paths as they propagate back through the PM fiber and EOM toward the polarizer. After passing the polarizer, the light is directed by the circulator to an AC-coupled photodetector. Because the two main beams traverse identical optical paths from source to detector—apart from the phase shift induced by the sample—the setup is sensitive solely to the TRS-breaking signal. The same experimental setup for high-resolution Kerr rotation was used in Science [89]. In this paper, broken time-reversal symmetry in the heavy fermion superconductor UPt_3_ was observed.

**Figure 5:**
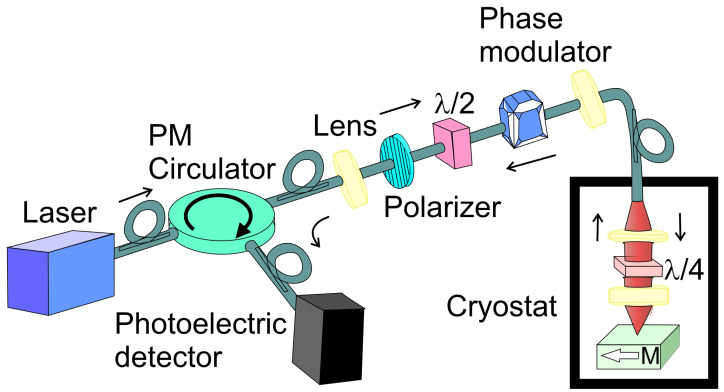
The experimental setup utilizes a Sagnac interferometer to measure phase shifts with high precision. Arrows show the direction propagation of light. PM circulator—A Polarization Maintaining Optical Circulator. Inspired by [86].

A next-generation experimental setup utilizing high magnetic fields is described in [90]. The optical system is similar to that shown in Figure 5. The authors employ a pulsed magnetic field apparatus capable of generating field strengths beyond the reach of conventional DC magnets. A major challenge in such experiments lies in the limited range of compatible measurement techniques, due to the short duration of the field pulses and significant electromagnetic and mechanical noise. To address this, the study introduces a novel approach for performing polar MOKE measurements under pulsed magnetic fields with a pulse width of 2 ms. This method integrates a compact, ferrule-based sample–fiber mounting system with phase-resolved numerical lock-in detection, and it employs a high-resolution, all-fiber loop-less Sagnac interferometer. Using this setup, the authors successfully measured MOKE signals in various ferromagnetic and ferrimagnetic materials at fields exceeding 40 T and temperatures down to 77 K, significantly extending the capabilities of pulse-field MOKE instrumentation reported in prior work.

An alternative approach has been introduced to enhance the sensitivity of T-MOKE measurements [91]. The optical setup is similar to that shown in Figure 4; however, the optical elements such as the QWP, polarizer, and analyzer are motorized. This method involves a specifically designed detection scheme in which the polarization conditions are systematically varied throughout the measurement process. This methodology allows for the identification and separation of noise and spurious background signals originating from the sample, the surrounding environment, or the detection system itself. The described experimental configuration employs an ultra-low-noise laser operating at a wavelength of 635 nm, with an incidence angle of 60° relative to the surface normal. The laser beam is initially linearly polarized by a polarizer, then directed onto the sample positioned within the gap of an electromagnet. After reflection from the sample surface, the light passes through a rotatable QWP and a second rotatable linear polarizer, before the transmitted intensity is recorded by a photodetector. The incident polarization is defined by the angle of P1, which was fixed at 45° relative to the plane of incidence, based on prior findings indicating that this setting maximizes signal strength and the signal-to-noise ratio in effective polarization T-MOKE measurements.To enable fine control over the spacing between the magnet and the sample, the magnet is positioned on a linear stage. The optical system responsible for preparing the incident beam and collecting the reflected beam is mounted on separate arms of the ellipsometer. By isolating these artifacts from the genuine T-MOKE response, the method substantially enhances both the sensitivity and reliability of the measurement, leading to unprecedented levels of performance [91].

This ellipsometer setup builds on foundational contributions from earlier researchers in the field [92,93,94]. The described configuration exhibits polarization sensitivity, as the detected light intensity varies with the applied magnetic field, reflecting changes in the sample’s magnetization state. In earlier T-MOKE investigations [95], where the orientations of P1, QWP, and P2 were held constant, the measured intensity difference ΔI between magnetization-reversed states was found to be proportional to the ellipticity induced by the T-MOKE effect [96,97]. In another study, the focus was on the surface magneto-optic Kerr effect in different materials [96]. The measurement system was also based on an ellipsometer. A distinctive feature of this setup is the use of a Faraday modulator in combination with lock-in detection to minimize noise. The lock-in detection and polarization modulation become particularly valuable when performing simultaneous magneto-optical measurements of Kerr rotation and ellipticity across a range of wavelengths. An example of this methodology can be found in the work by Osgood et al. [98]. In a typical setup, light from a lamp source is directed through a monochromator, a polarizer, and a photoelastic modulator operating at 50 kHz, before being focused onto the sample surface. Upon reflection, the light acquires both magneto-optic rotation and ellipticity. The reflected beam is then expanded by an output lens and passes through an analyzing polarizer set at 45° relative to the input polarizer. The resulting beam is detected by a photodiode. The angle of incidence is 36° from the surface normal. The DC and AC components of the detected signal are separately amplified and fed into a divider circuit, which is connected to the input of a lock-in amplifier [98]. A different approach for measuring the refractive index using the Kerr effect has been explored. This straightforward goniometric technique yields results that align well with established literature values [99].

General overviews of polarization modulation and related optical techniques are available in standard references [100,101,102,103,104], while the effects of imperfections in the polarizer and analyzer in a magneto-optical Kerr spectrometer have been addressed in recent analyses [105]. Therefore, assuming an extinction ratio of 0.01 for the polarizer, the typical measurement error is approximately 1%. This simplified estimate is based on the assumption that the Kerr rotation and ellipticity of the sample are of comparable magnitude [105].

Additionally, polarization modulation has been applied in in situ MOKE studies to determine the Curie temperature of Gd thin films [61].

The investigation of the SMOKE requires significantly higher precision and more accurate measurement techniques compared to bulk MOKE studies [106]. This is primarily due to the fact that SMOKE signals originate from a very thin interfacial region—often just a few atomic layers—making them inherently weaker and more susceptible to noise and surface contamination. As a result, experimental setups must maintain sub-nanometer surface quality and high signal-to-noise ratios, often operating under high-vacuum conditions and utilizing synchrotron radiation or other highly coherent light sources to reliably detect the magneto-optical response confined to the surface.

As noted in [96], lock-in detection techniques have generally not been emphasized in the context of SMOKE, since modern computer-controlled setups can achieve sufficient signal averaging to enhance the signal-to-noise ratio. An early SMOKE study [107] even demonstrated that hysteresis loops acquired via lock-in detection of the photodiode signal—using a commercially available photoelastic modulator to modulate the polarization of the incident beam—showed no improvement over results obtained with a straightforward DC measurement approach. In the scientific article by Genuzio et al., a custom-designed, ultra-high vacuum (UHV)-compatible MOKE magnetometer is presented for applications in surface science and materials research. The system operates in conjunction with a Photoemission Electron Microscope (PEEM) at the Nanospectroscopy beamline of the Elettra synchrotron [108,109,110,111,112,113,114,115].

A Kerr rotation measurement system designed for use in pulsed magnetic fields up to 11 T was developed to investigate the magnetic properties of ultrathin (Fe, CoFeAl, MnAs and CoMnAl) films [80]. The setup utilizes a narrow-bandwidth (ca. 2 nm) quasi-monochromatic light source derived from a NPCF (nonlinear photonic-crystal fiber) laser (500–900 nm). Wavelength selection is achieved using a polychromator(a delta prism, an adjustable aperture, and an electronically controlled mirror). Polarization control is implemented using two Glan–Taylor prisms: one fixed as a polarizer before the sample, and the other mounted on a mechanical rotator to vary the polarization plane. The light beam is focused to 500 μm on the sample surface. The reflected light is analyzed using a balanced optical bridge detector to measure the differential intensity, corresponding to the Kerr rotation. A 7% reflective glass provides a reference signal. The pulsed magnetic field is generated by a custom-built coil powered by a capacitor bank, producing short-duration fields oriented perpendicular to the sample surface. Data acquisition runs at 1 MS/s, capturing magnetic field, reflectance, and Kerr signal simultaneously.

A similar configuration with low magnetic fields in optical set up was used to detect the asymmetric magnetization reversal in exchange biased Fe/MnF_2_ [116], FeF_2_ [117] and Co/CoO thin-film systems [118]. In these papers, Kerr loop measurements were conducted using two distinct geometrical configurations. In the first, referred to as the horizontal configuration, the plane of light incidence lies parallel to the sample’s longitudinal magnetization M_*L*_ characteristic of the longitudinal Kerr effect. In contrast, the vertical configuration aligns the incidence plane with the transverse magnetization M_*T*_, enabling sensitivity to the transverse Kerr effect. In both setups, the polarization state of the incident laser beam (initially linearly polarized) can be continuously tuned between s- and p-polarization states via a HW retardation plate. Kerr rotation of the reflected light was detected concurrently in both configurations. The reflected beam was directed a balanced polarimeter and focused onto photodiodes. The individual intensities at each diode as well as their differential signal were recorded simultaneously using a lock-in amplifier. Prior to each measurement, the diode bridge was calibrated by adjusting the HWP to ensure a balanced condition.

**Figure 6:**
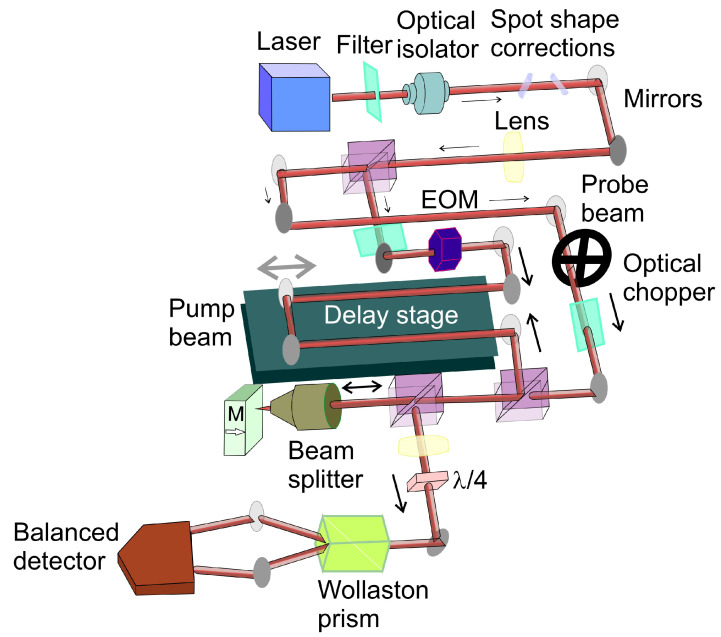
Advanced optical setup with a pump–probe configuration, light modulation, and a balanced polarimeter. EOM—electric optical modulator, optical chopper connected to Lock-in amplifier. Black arrows show the direction propagation of light. The gray arrow indicates the movement of the delay stage. Inspired by [119].

An additional noteworthy experimental setup is illustrated in Figure 6. Comprehensive details regarding this setup or similar are provided in many papers [119,120,121,122,123,124,125,126]. In time-resolved magneto-optical Kerr effect (TR-MOKE) measurements, a mode-locked Ti:sapphire laser generates ultrafast pulses (≈100 fs duration) centered around 800 nm. The beam is split into orthogonally polarized pump and probe components using a polarizing beam splitter. A delay stage modifies the optical path length of the pump beam, enabling precise control of the time delay (up to 4 ns) between excitation and probing. Both beams are focused onto the sample surface, with spot sizes ranging from several hundred nanometers to tens of micrometers. The reflected probe beam is then separated into orthogonal polarizations by a Wollaston prism, and the intensity difference between these components is measured by a balanced detector. These changes are directly linked to the probe beam’s polarization rotation, which reflects magnetization dynamics in the sample via the Kerr effect. At early time delays (Δt < 10 ps), the TR-MOKE signal is dominated by nonequilibrium thermalization processes involving electrons, magnons, and phonons. This regime can be modeled using a phenomenological three-temperature framework describing energy exchange among these subsystems (Te, Tm, Tp). At later delays (hundreds of ps), the signal exhibits damped oscillations due to spin precession, allowing extraction of magnetic damping parameters.

Additionally, pump–probe techniques can be extended to transmission-mode configurations—particularly for (semi-)transparent samples—enabling measurement of Faraday rotation instead of Kerr rotation.

Nearly identical pump–probe experiments were conducted also to confirm the Kerr-type origin of the nonlinear response in paper [127]. An incident pulse was split into pump and probe beams, with the probe delayed by a variable optical delay and attenuated by a factor of 100 to isolate pump-induced reflectance changes. Both beams were focused onto the sample, with the probe spot kept significantly smaller to ensure uniform excitation. The probe remained s-polarized, while the pump polarization was rotated by 90 deg to suppress coherent artefacts such as the coherence spike near zero delay. The reflected probe was detected by a photodiode with polarization filtering to minimize scattered light. The pump was mechanically modulated, and the probe reflectance modulation was measured using lock-in detection to enhance sensitivity. Linear reflectance was measured independently, and all measurements were performed at intensities approximately 15% below the damage threshold [127]. In such setups, numerous studies have reported a pronounced peak near zero time delay—referred to as a coherence spike (also called coherent interaction or coherent nonlinearity)—in femtosecond pump–probe measurements [128,129,130,131]. This effect must be carefully considered, as it can introduce artifacts into pump–probe data that may be mistakenly interpreted as an intrinsic sample response.

**Figure 7:**
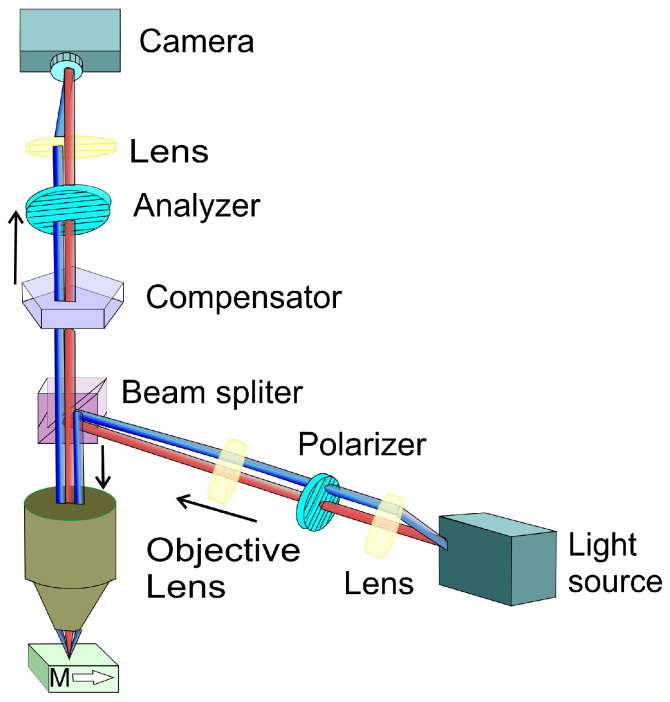
Scheme of MOKE microscope. The translation plate can be mounted on a biaxial goniometer in order to displace the laser beam along two perpendicular directions by varying the angles δ and β. Black arrows show the direction propagation of light. Inspired by [132].

Another group of measurement devices are Kerr microscopes [132,133,134]. Figure 7 shows the schematic of the experimental equipment layout. Kerr microscopy remains an indispensable technique for investigating spin system dynamics, magnetic domain configurations, and emergent magnetic quasiparticles such as skyrmions. Recent advancements have expanded its relevance to cutting-edge research areas, including antiferromagnetic spintronics, magneto-plasmonics, and studies of two-dimensional and topological materials. These developments are particularly oriented toward enhancing the spatial, temporal, and spectral resolution of the technique [119,123,135,136,137]. Furthermore, emerging progress in the field of topological insulators has the potential to reinvigorate interest in MOKE-based memory technologies, offering new avenues for non-volatile, optically addressable data storage [138,139]. A modified three-lock-in amplifier system was employed to describe a new approach to scanning MOKE microscopy [140]. Image acquisition is typically performed using a CCD camera interfaced with object the oriented micromagnetic framework ver.1.0. for data processing and analysis. Depending on the chosen optical configuration, Kerr microscopy enables the detection of different magnetization vector components: either the in-plane magnetization (using longitudinal or transverse MOKE geometries) or the out-of-plane component (via polar MOKE geometry).

A novel approach was developed to effectively suppress parasitic Faraday contributions in wide-field polarization microscopy, even under arbitrary measurement conditions [141]. This was achieved by integrating a motorized analyzer into the optical path of the microscope and continuously monitoring the live image brightness. By dynamically adjusting the analyzer to maintain a constant brightness level, the method enables high-fidelity imaging of polar magnetic domains across the full accessible magnetic field range. To isolate the MOKE signal from unwanted Faraday contributions, non-magnetic regions within the sample were used as reference points for brightness stabilization. Its utility was demonstrated in complex multilayer thin-film systems eg. FePd/FePt/FePd and FePt.

### 3.2. Electro-Optical Kerr Effect

Since the 1990s, the use of the electro-optic Kerr effect with alternating current (AC) field modulation has steadily gained recognition as a robust technique for electric field measurement in dielectric liquids [142,143]. As a result, numerous research groups have enhanced and optimized this technique. For instance, the authors’ research team has developed a non-contact, temperature-regulated platform for electric field measurements in insulating oils, providing a practical reference for designing systems to monitor electric fields in oil-immersed capacitors [144,145,146,147].

The underlying principle of the electro-optic Kerr measurement lies in the field-induced birefringence observed in isotropic, transparent dielectric liquids. When an external electric field is applied, the material exhibits optical anisotropy, introducing a phase shift between the components of light polarized parallel and perpendicular to the field direction. This induced birefringence results in a measurable phase difference, which is directly related to the magnitude of the applied electric field. Generally, in major experimental setups, it comprises the following key optical and electronic components: Linearly polarized light source, typically a helium-neon laser emitting at 632.8 nm, which was chosen for its superior beam quality and stability, ensuring reliable optical input for the system. To prepare and analyze polarized light, a polarizer and analyzer were employed. A typical Glan–Taylor polarizer with an extinction ratio exceeding 10^5^ was selected to meet the high-precision requirements of the experiment. A zero-order quartz QWP was used to convert linearly polarized light into circularly polarized light with minimal phase error. A photodetector was used to convert the optical signal into an electrical signal, which could then be analyzed. A lock-in amplifier was employed to isolate and extract the fundamental and harmonic signal components corresponding to the modulated electric field [148].

**Figure 8:**
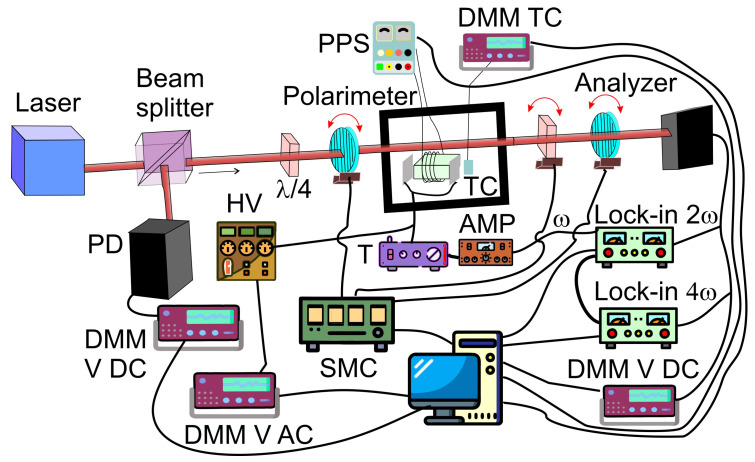
Schematic diagram of the measurement system of EOKE. PPS—a programmable power supply, DMM TC—a digital multimeter with thermocouple, HV—a high voltage power supply, T—a transformer, AMP—an amplifier, PD—a photo-diode, DMM V DC—a digital multimeter DC voltage, DMM V AC—a digital multimeter AC voltage, SMC—a stepper motor. Black arrow shows the direction propagation of light. The red arrow indicates the rotation of the optic element. Inspired by [149].

A novel implementation of the dynamic polarimetric technique has been presented by Ledzion et al. [149]. The system depicted in Figure 8 was used to carry out electro-optical measurements. Proposed configuration enables the separation of electro-optic Kerr effect contributions from those caused by electro-absorption in semi-transparent liquid media.

The experimental sample consisted of oil contained with stainless steel electrodes. The electrodes, were spaced at a fixed distance by Teflon spacers. A He-Ne laser (632.8 nm) served as the coherent light source. The laser beam was split by a semi-transparent mirror into two paths. The reflected beam was directed toward a photodetector, which was connected to a DC voltmeter to measure the reference input voltage. The transmitted beam, after passing through the semi-transparent mirror, was routed through an electro-optical modulator before reaching a second photodetector. The output signal from this detector was decomposed into its DC component measured with another multimeter, and its AC components at the second and fourth harmonics of the modulation frequency. These harmonic components were captured using two lock-in amplifiers. Although the fourth harmonic was not used in the quantitative analysis, its measurement confirmed the negligible contribution of higher-order and mixed-frequency terms. The modulation signal itself was a sinusoidal waveform generated by one of the lock-in amplifiers. This signal was amplified using an amplifier and subsequently stepped up by a high-voltage transformer. The voltage at the transformer output was monitored using a high-voltage probe connected to an AC voltmeter. The electro-optical modulation assembly comprised a polarizer, the oil sample placed between the electrodes, a quarter-wave plate, and an analyzer. To ensure thermal stability, the cuvette and electrode assembly were housed within a temperature-controlled measurement chamber, maintaining an accuracy of ±0.2 °C. Precise angular control of the polarizer, quarter-wave plate, and analyzer was achieved by mounting each on a nanorotation stage.

### 3.3. Optical Kerr Effect

Recent advances in nonlinear optics have led to a renewed interest in measuring higher-order Kerr effects in various optical materials. Among these, diamond has emerged as a particularly promising platform due to its exceptional thermal conductivity, wide bandgap, and ability to host optically active color centers such as negatively charged nitrogen-vacancy (NV^−^) defects. The most recent studies have focused on quantifying the anisotropy of the nonlinear refractive index in diamond, using the Z-scan technique as a precise tool for probing optical Kerr effects under ultrafast laser excitation [83].

A representative example of such research explores the nonlinear optical response of diamond crystals doped with NV centers, with particular emphasis on the optical Kerr effect in the infrared spectral region. Using the Z-scan method with 230 fs laser pulses at a central wavelength of 1032 nm, both high-purity and NV-enriched diamond samples were investigated at NV concentrations of 0.3 ppm and 4.5 ppm, respectively. The experimental results reveal a pronounced anisotropy in the nonlinear refractive index, exhibiting a four-fold rotational symmetry, the amplitude of which decreases with increasing NV concentration. These findings provide valuable insights into how defect engineering can be used to control nonlinear optical properties in wide-bandgap crystals.

**Figure 9:**
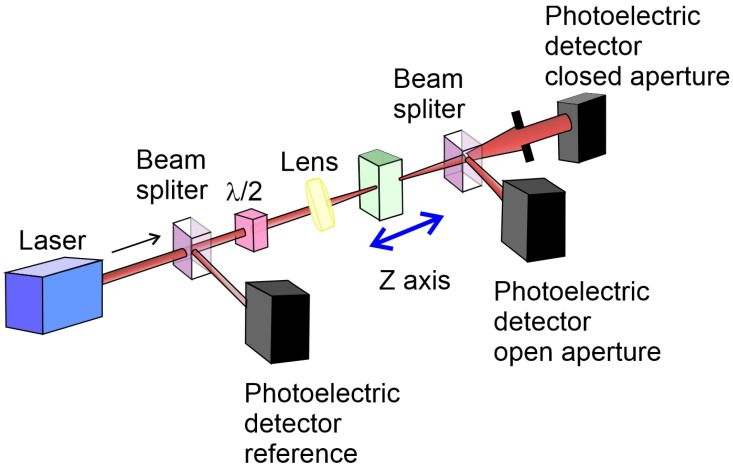
Schematic diagram of the measurement system of the third-order optical KE. The crystal mounted on a translation stage. The setup also features two detectors: an open-aperture (OA) detector and a closed-aperture (CA). The black arrow shows the direction propagation of light. The blue arrow indicates the movement of the motorized stage. Inspired by [83].

The experimental configuration (illustrated in Figure 9) follows a standard Z-scan setup and allows measurements in both closed-aperture (CA) and open-aperture (OA) modes, enabling the simultaneous evaluation of nonlinear refraction and nonlinear absorption, respectively. The light source was a Yb-fiber laser generating 230 fs pulses at 1032 nm and a maximum pulse energy of 10 µJ. A portion of the beam was diverted by a beam splitter to monitor pulse stability, while the transmitted portion was focused onto the sample using a lens with a focal length of 10 cm, producing a focal spot diameter of $2w_{0}=70\;\mu m$ and a corresponding Rayleigh range of $2z_{0}=7.3$ mm.

The transmitted light in both OA and CA modes, as well as a reference signal, was detected using identical large-area photodiodes connected to a digital oscilloscope. The OA and CA signals were normalized to the reference signal, and the normalized data were averaged over 500 laser shots before being transferred to a computer for further analysis. This averaging procedure significantly enhanced the signal-to-noise ratio, thereby improving the accuracy and reproducibility of the results.

One of the inherent limitations of the Z-scan technique in CA mode arises from the lateral movement of the transmitted laser beam across the aperture as the sample is translated along the z-axis. This beam wander can degrade the precision of the measurement and reduce sensitivity, particularly when small nonlinearities are being investigated. To compensate for this effect, reference measurements were conducted at very low pulse energies—below 30 nJ (corresponding to 7 GW/cm^2^)—where nonlinear effects are negligible across all z-positions. The OA and CA curves recorded under these conditions were subsequently used to correct the corresponding high-intensity data, ensuring that only genuine nonlinear contributions were analyzed.

In another paper, a femtosecond laser was used as the light source. The beam was split into signal and gate pulses using a 50/50 beam splitter. The optical Kerr gate (OKG) consisted of crossed Glan–Taylor polarizers and a CS_2_ Kerr medium in a cuvette. The gate and signal pulses intersected at an 8° angle. The signal beam was focused into the Kerr medium by a 250 mm lens and collected by a 200 mm lens. Thicker Kerr media and smaller crossing angles increase interaction length but may affect switching time and scattering. This setup allows ultrafast modulation of the signal beam via the nonlinear optical Kerr effect. The polarization-based configuration ensures high contrast and precise temporal gating. Such arrangements are widely used in time-resolved spectroscopy and ultrafast optical measurements [150].

Furthermore, the potential polarization anisotropy of the nonlinear response was investigated by varying the polarization direction of the incident beam relative to the crystal axes. This was accomplished using a half-wave plate inserted in the laser path, enabling systematic characterization of the dependence of the Kerr nonlinearity on crystal orientation.

## 4. Kerr Effect in Ceramics

Kerr effect occurs in various materials. From our point of view, ceramic materials are the most interesting in this aspect. In this section, we briefly introduce groups of materials in which Kerr effect can be observed with specific effects detected and utilized in them. Transparency is a fundamental requirement for EO applications. The most conventional ferroelectric ceramics exhibit poor transparency for several intrinsic and microstructural reasons. At the molecular level, their perovskite lattice typically deviates from a cubic, optically isotropic phase, leading to anisotropic refractive indices and intrinsic light scattering. In addition, the grains in polycrystalline ceramics are randomly oriented, and their boundaries contain structural imperfections such as vacancies, dislocations, segregated impurities, and other defect complexes. These inhomogeneities disrupt the local polar order and act as scattering centers for incident photons. Furthermore, residual porosity within the ceramic body introduces air–solid interfaces with large refractive-index mismatches, which further intensify scattering and attenuate transmitted light.

To mitigate these issues and enhance optical transparency, targeted chemical doping has been widely employed. Extensive research has since focused on how lattice symmetry, grain size distribution, defect chemistry, and relaxor-type molecular dynamics influence both optical transparency and the electro-optic response of ceramics, particularly as a function of dopant concentration.

### 4.1. Cubic Materials

Cubic materials are characterized by a symmetric crystal lattice where all three axes are equal with angles of 90° between them. Such structure promotes light propagation without scattering and, indeed, cubic materials are among the best transparent materials. However, not only isometric structure is required for unhindered light propagation—a suitable electronic structure is also required. Materials with small band gap will strongly absorb visible light. Electronic transitions such as d-orbital transition in transition metals may also hinder light propagation. For these reasons, cubic materials such as PbS and Cu_2_O are not transparent. Moreover, defects, rough surfaces, impurities, pores or grain boundaries may decrease transparency of materials significantly by scattering or absorption of light in polycrystalline materials.

A more detailed molecular perspective shows that optical transparency in cubic materials is tightly controlled by the electronic configuration and the character of chemical bonding between constituent atoms. Highly ionic or predominantly σ -type covalent bonds typically produce wide band gaps due to strong localization of valence electrons, which suppresses electronic transitions within the visible range [151,152]. In contrast, systems exhibiting significant p–d or d–d hybridization often possess narrower band gaps or enable parity-allowed ligand–field transitions that introduce additional absorption bands [153,154]. The symmetry inherent to cubic lattices influences the splitting of electronic energy levels, particularly in octahedral or tetrahedral coordination environments, and can therefore modulate the optical response. Defects such as vacancies, interstitials, or substitutional impurities create localized electronic states within the band gap, acting as color centers capable of absorbing specific wavelengths of light. Bond polarizability and the spatial distribution of electron density further affect the refractive index by altering the amplitude of electronic polarization under an applied electromagnetic field [155,156]. Consequently, transparency arises from the combined effects of crystal symmetry, electronic structure, bonding characteristics and defect chemistry rather than from geometric arrangement alone.

#### 4.1.1. Spinels

Spinels are the group of compounds with the general formula of AB_2_X_4_, where A (divalent cations) may be Mg, Fe, Mn or Zn, B (trivalent cations) may be Fe, Mn, Al, or Cr, and the X anion may be O, S, Se, or Te [157,158]. An intermediate arrangement between normal and inverse spinels is mixing spinels with varying degrees of cation site occupancy. Bragg was one of the first researchers who described the structure of spinels in 1915 [157], commenting:

*“It is very interesting that a somewhat complicated composition should be associated with such complete crystalline symmetry.”*

The majority of materials in this group belong to the space group Fd3m. In spinels, the primitive tetrahedral cell is made up of two AB_2_X_4_ formula units. When four of these primitive cells are combined, they form the conventional cubic unit cell. From a molecular standpoint, the properties of spinel structures arise from the specific distribution of A and B cations between tetrahedral and octahedral sites of the lattice. The degree of inversion—defined as the fraction of trivalent cations occupying tetrahedral positions—strongly influences electronic structure, bond covalency, and ultimately the optical, magnetic, and transport properties of the material.

At the electronic level, the crystal field environment imposed by the tetrahedral (A) and octahedral (B) coordination sites splits the d orbitals of transition-metal cations into energetically distinct subsets. This splitting modulates the energies of d–d transitions, charge-transfer excitations, and hybridization between metal d states and p orbitals of the anions. As a result, variations in cation occupancy lead to measurable differences in band gap, polarizability, and local symmetry-induced selection rules governing optical absorption.

The molecular bonding framework in spinels is further governed by the interplay between ionic and covalent contributions. Stronger p–d hybridization—commonly observed in sulfide, selenide, and telluride spinels—results in narrower band gaps and enhanced optical absorption compared to their oxide analogues. Conversely, oxygen-based spinels exhibit more ionic bonding, wider band gaps, and reduced electronic polarizability. Differences in the electronegativity of the anion X also affect the energy and dispersion of valence bands, thus modulating the refractive index and overall optical response.

The nature of the X anion also plays a noticeable role in shaping the bonding inside the spinel lattice. Sulfide, selenide, and telluride spinels generally show stronger p–d hybridization than oxides, which tends to narrow their band gaps and increase their electronic polarizability. As a result, these compounds often display enhanced nonlinear optical behavior compared to their oxide counterparts. Oxide spinels, on the other hand, are more ionic in character and therefore usually have wider band gaps and lower $\chi^{\left(3\right)}$, although they benefit from better overall transparency. Changes in the electronegativity of the anion shift the energy and dispersion of the valence bands and this in turn affects properties such as refractive index, the position of the absorption edge, and the strength of magneto-optical responses.

The main representative of the group is MgAl_2_O_4_, a mineral spinel. Along with AlON, which is also characterized by cubic structure, they were among the first sintered transparent ceramics. Magnesium aluminate is has excellent optical properties with a large range of transparency (0.4–0.5 μm) and good mechanical properties. Those characteristics makes it an attractive candidate for applications such as transparent armors [159].

AlON, on the other hand, is characterized by a broader range of transparency (0.2–6.0 μm) but is typically less transparent in visible range due to intrinsic absorption [160], thus performing better in long IR-range in comparison to MgAl_2_O_4_.

Defect chemistry is another crucial molecular aspect decidet about transparency and lattice structure. Antisite defects, vacancies, and cation disorder introduce localized electronic states within the band gap, which can act as color centers or sites for electron–phonon coupling. Such intrinsic or extrinsic defects influence phonon spectra, scattering processes, and absorption characteristics. Because the spinel structure tolerates a considerable degree of cation substitution, the resulting configurational disorder can significantly alter optical transparency and electronic conductivity, especially in mixed-metal or non-stoichiometric compositions.

#### 4.1.2. Garnets

Garnets are another type of materials with cubic structure that is of great importance in the field of transparency. They have the typical chemical formula of A_3_B_2_C_3_O_12_. In the garnet structure, the A-site cation occupies a dodecahedral coordination environment, whereas the B-site cation is situated in an octahedral site, and the C-site cation is tetrahedrally coordinated. The A position is typically filled by relatively large cations, such as Y^3+^, Lu^3+^, Gd^3+^, Tb^3+^, Ca^2+^, or Sr^2+^. In contrast, the C position is usually occupied by cations like Si^4+^, Ge^4+^, Al^3+^, or Fe^3+^. The garnet framework accommodates a wide range of compositional substitutions, giving rise to numerous solid-solution series. This compositional flexibility makes garnets an important subject of materials science research, particularly in efforts to tailor their properties for targeted applications [161,162,163].

From a molecular standpoint, the garnet structure is defined by a network of BO_6_ octahedra and CO_4_ tetrahedra linked through corner sharing, with A cations positioned in the interstitial cages. Variations in cation species and valence introduce subtle changes in local coordination geometry, p–d hybridization strength, and the distribution of crystal-field environments. These factors influence band-edge positions, electronic polarizability, and the energy of charge-transfer and d–d transitions—properties directly relevant for both linear and nonlinear optical behavior, including magneto-optical effects.

Garnets can be magnetic like their flagship representative, Y_3_Fe_5_O_12_ (YIG) which is ferrimagnetic [8,161]. The magnetic character of YAG, Tb_3_Ga_5_O_12_ and other garnets makes it possible to observe Faraday rotation in them. Garnets are often used in lasers and scintillators (for high-energy photons conversion). Gadolinium gallium garnet (Gd_3_Ga_5_O_12_, GGG) is another prominent example of garnets. Its properties and improving them are often of interest to researchers, including doping and the influence of microstructuring it [164].

Inclusions of other elements in garnets give them color (pure garnets are typically colorless), and they can be used as gemstones.

#### 4.1.3. Sesquioxides

Another important class of transparent ceramic materials is sesquioxides, compounds with the general formula of A_2_O_3_. Their name derives from the Latin prefix “sesqui-”, meaning “one and half” which corresponds to the ratio of oxygen to metal atoms in their molecule (in contrast to monoxides, AO, or dioxides, AO_2_). Composed typically of trivalent metal cations, such as Y^3+^, Al^3+^, Lu^3+^, or Sc^3+^ (the “A” component), these oxides posses exceptional mechanical and optical properties such as high optical transparency across a wide spectral range, from ultraviolet to the infrared.

The optical and electronic properties of sesquioxides are largely determined by the arrangement of A cation within the oxide lattice and the nature of their bonding with oxygen.

Among sesquioxides, Al_2_O_3_ (γ-Al_2_O_3_ crystallizes in cubic structure) and Y_2_O_3_ are the most studied representatives of the group. The coordination environment of Al^3+^ cations and the degree of covalency in Al-O bonds influence band gap energy, refractive index, and the likelihood of nonlinear optical processes. Similarly, Y^3+^ ions in Y_2_O_3_ can occupy two distinct crystallographic sites, causing subtle differences in crystal-field splitting that be utilized for dopant-related optical functionality.

In order to further expand the versatility of sesquioxides, doping is often used with elements like Er^3+^ [165] or Yb^3+^ [166]. These dopants introduce localized electronic states within the band gap. The dopants interact with the host lattice through f-d or f-f transitions, and the resulting spin–orbit coupling and crystal field effects can modify absorption, emission, and Faraday and Kerr effects occurring in the materials.

#### 4.1.4. Fluorides

Because of their simple crystal chemistry and high purity achievable during synthesis, fluoride-based ceramics also serve as valuable group of transparent materials. Fluorides, with the representatives like CaF_2_, MgF_2_, SrF_2_, and LiF, are characterized by low refractive indices, minimal optical dispersion, and good chemical inertness and are often utilized in laser optics [162,167], infrared windows [167], and ultraviolet transmission components [168,169].

The transparency and optical properties of fluorides arise from the strongly ionic nature of metal–fluoride bonds, resulting in wide band gaps and very low electronic absorption across the ultraviolet, visible, and infrared spectral regions. The simple cubic or tetragonal lattices of these materials provide well-defined, defect-minimized environments that reduce scattering and enhance optical clarity. Minor defects, such as F^−^ vacancies or cation substitutions, can locally disturb the lattice, producing color centers or affecting nonlinear optical responses, but high-quality synthesis can largely suppress these effects.

#### 4.1.5. Sulfides and Selenides

Finally, there exist prominent chalcogenide materials prized for their wide transmission in the mid- to long-wave infrared (MWIR, LWIR) bands and compatibility with high-power and thermal imaging systems. In particular, zinc sulphide, ZnS, offers high transition in the 7–12 μm region and allows useful transmission down toward the visible range in its multispectral form [170]. Recent works also demonstrated denser polycrystalline doped ZnS fabricated by spark plasma sintering with reduced scattering loss making it more practical for IR optics [171].

Zinc selenide, ZnSe, provides even broader IR transmission (approximately 0.6–15 μm) and good performance under thermal loads [172].

The optical properties of these chalcogenides are governed by the combination of covalent and ionic bonding between the metal cations and the chalcogen anions. Stronger p–d and s–p hybridization in sulfides and selenides lowers the band gap compared to oxides, enabling extended IR transparency and higher electronic polarizability. These hybridizations also influence nonlinear optical susceptibilities and contribute to the robustness of their refractive indices across wide spectral ranges.

#### 4.1.6. Perovskites

Perovskites, with the general formula of ABX_3_, constitute a versatile class of transparent ceramics.

The A-site cations, occupying the cube corners of the perovskite structure, are typically large alkaline-earth metals (Ca^2+^, Sr^2+^, Ba^2+^), rare-earth metals (La^3+^, Sm^3+^, Nd^3+^) and sometimes monovalent cations for halide perovskites: Cs^+^, Rb^+^ and K^+^. Those cations provide lattice stability and affect lattice constant and tolerance factor. A good size match promotes cubic symmetry which favors isotropic optical properties and transparency.

The B-site cations occupy the body center coordinated by six X anions forming octahedra, BX_6_. They are usually smaller than A-cations and are often main group metals (Al^3+^, In^3+^, Ga^3+^), transition metals (Ti^4+^, Ta^5+^, Zr^4+^, Nb^5+^), or chalcogenides in halide or sulfide perovskites (Sn^2+^ or Pb^2+^). The electronic configuration of B-site cations strongly influences band structure, band gap, and optical transparency, and, in magnetic perovskites, can induce significant magneto-optical effects such as Faraday and Kerr rotation.

The X-anion is usually O^2−^ (oxide perovskites) but can also be halides (F^−^, I^−^, Br^−^, Cl^−^) or chalcogenides (S^2−^, Se^2−^) in halide and chalcogenide perovskites, respectively. The nature of the X anion modulates p–d hybridization with the B-site cations, affecting electronic polarizability, band dispersion, and nonlinear optical responses. Stronger covalent interactions in chalcogenide perovskites reduce the band gap and extend transparency into the infrared, whereas oxide perovskites exhibit wider band gaps, higher chemical stability, and generally lower optical losses.

This structure allows for wide variety of cation combinations for fine tuning electronic, optical, and mechanical properties.

Oxide perovskites, such as SrTiO_3_, BaTiO_3_, and LaAlO_3_ are known as good transparent ceramic materials due to their high refractive indices, wide optical windows (visible to near-infrared), and good thermal and chemical stability.

Magnetic perovskites, including those containing Fe, Co, or Mn at the B site, additionally enable magneto-optical phenomena, linking their atomic-scale electronic structure to observable Faraday and Kerr effects.

### 4.2. Non-Cubic Anisotropic Materials

Transparency is not exclusive for only cubic materials. Cubic crystals usually show optical isotropy due to their high symmetry, but many non-cubic materials can also be transparent, though their optical characteristics tend to be more intricate. In lower-symmetry structures—like tetragonal, hexagonal, orthorhombic, monoclinic, or triclinic crystals—the refractive index typically varies with direction. This leads to optical anisotropy, where light traveling along different crystallographic axes encounters varying optical effects. A prominent example of anisotropy in non-cubic transparent materials is birefringence, where a single incoming light beam divides into two rays with distinct velocities and polarizations.

#### 4.2.1. Hexagonal and Rhombohedral Materials

Among transparent ceramics, several non-cubic systems, such as Al_2_O_3_ and Si_3_N_4_, are of particular interest because of their optical transparency combined with high mechanical and thermal stability.

Al_2_O_3_, in its most stable α-phase (corundum), crystallizes in a rhombohedral structure belonging to the trigonal crystal system (space group $R\bar{3}c$)—it can also be described in a hexagonal representation. Despite being anisotropic and birefringent, dense polycrystalline α-Al_2_O_3_ can still exhibit high optical transparency in the visible and near-infrared ranges, provided that grain sizes are small enough and residual porosity is kept minimal. At the atomic level, the anisotropy arises from the asymmetric stacking of oxygen and aluminum layers along the c-axis, which induces direction-dependent electronic polarizability and refractive indices.

In contrast, Si_3_N_4_ occurs mainly in α- and β-polymorphs, both of which belong to hexagonal crystal systems (space groups P3_1_c and P6_3_/m, respectively). These phases are also optically anisotropic, yet they remain highly transparent in the near-IR region when sintered to full density with as few scattering centers as possible. The optical anisotropy of Si_3_N_4_ is linked to its hexagonal arrangement of SiN_4_ tetrahedra, which produces direction-dependent electronic transitions and local polarizability variations.

The optical performance of such non-cubic ceramics is often compared with cubic transparent materials like Y_2_O_3_ or MgAl_2_O_4_ spinel, which are isotropic and therefore free from birefringence effects.

#### 4.2.2. Tetragonal and Monoclinic Materials

ZrO_2_ is another important transparent ceramic, notable for its multiple polymorphs, including tetragonal and monoclinic phases, which offer a unique combination of optical transparency, high toughness, and thermal stability. Tetragonal ZrO_2_, often stabilized at room temperature with Y_2_O_3_ (YSZ), crystallizes in a tetragonal system (space group P4_2_/nmc) that is slightly distorted from cubic symmetry. When fully densified and with careful control of grain size, tetragonal ZrO_2_ can achieve significant transparency in the visible and near-infrared regions, although some light scattering or birefringence may occur due to lattice anisotropy. Monoclinic ZrO_2_, the stable phase at room temperature in undoped material, adopts a monoclinic structure (space group P2_1_/c) and is optically anisotropic. Achieving fully transparent ceramics in the monoclinic form is more challenging because phase transformations during cooling can generate microcracks and scattering centers. Nevertheless, high-quality polycrystalline zirconia, particularly in the tetragonal or partially stabilized forms, can combine transparency with excellent mechanical properties, making it suitable for optical windows, dental ceramics and other high-strength applications.

The optical anisotropy of ZrO_2_ can be explain at the molecular level and originates from the arrangement of Zr^4+^ cations within distorted oxygen coordination polyhedra. In the tetragonal phase, small deviations from cubic symmetry produce direction-dependent electronic polarizability, influencing the refractive index and nonlinear optical responses. The monoclinic phase exhibits more pronounced anisotropy due to asymmetrical Zr^4+^ coordination and tilting of oxygen octahedra, which affect local electronic transitions and light–matter interactions. Additionally, oxygen vacancies and Y^3+^ dopants in YSZ can introduce localized electronic states, impacting Kerr and Faraday effects as well as light absorption and scattering at the microscopic scale. These molecular-level features offer opportunities to tailor the optical, electronic, and magneto-optical properties of zirconia for advanced transparent ceramic applications.

### 4.3. Magneto-Optical Kerr Effect in Materials

Cerium antimonide, CeSb, is the material researcher observed the largest magneto-optical Kerr rotation. Firstly, in 1986, Reim and others reported the largest at that time observed value of more than 14° [173]. Ten years later, Pittini et al. reported an astounding 90° Kerr effect in the same material [174].

In 2018, Kocsis et al. reported a strong magneto-optical Kerr effect in the cubic (at room temperature) chromite spinel oxides FeCr_2_O_4_ and CoCr_2_O_4_ [175]. They observed the effect at the on-site *d-d* transitions of the tetrahedrally coordinated Co^2+^ and Fe^2+^ magnetic ions, and noted that the Kerr rotation of $\vartheta_{Kerr}\approx 12^{{}^{\circ}}$ in CoCr_2_O_4_ at ∼0.78 eV—corresponding to 1.55 μm, a telecommunications wavelength—is the largest reported for magnetic semiconductors and insulators. Building on that experimental highlight, Hossain et al. used both spin- and non-spin-polarized density functional theory to simulate the electronic and optical properties of CoFe_2_O_4_; their computed energy-loss spectra likewise indicate a large magneto-optical Kerr effect [176].

Experimental studies have since explored how growth mode and crystal quality affect MOKE. For example, Himcinschi and colleagues grew epitaxial, ordered arrays of CoFe_2_O_4_ and NiFe_2_O_4_ on Nb-doped SrTiO_3_ substrates by pulsed layer deposition and found MOKE spectra similar to those of single crystals of the corresponding compounds [177]; the nickel-based spinel showed roughly three times lower signal intensity. Earlier work by Peeters and Martens examined polar Kerr rotation in polycrystalline CoFe_2−x_Al_x_O_4_ (0 ≤x≤ 0.8) and Co_x_Fe_3−x_O_4_ (0 ≤x≤ 1.0) across 0.6–5.5 eV and concluded that magneto-optic responses of cobalt ferrites below 2 eV are governed not only by crystal-field transitions of Co^2+^ but also by a transition they assign to charge transfer from Co^2+^ to Fe^3+^ on octahedral sites [178].

Comparative studies help place these effects in context. Kim and coworkers compared bulk Fe_3_O_4_ (sintered pellets) with spin-coated CoFe_2_O_4_ and NiFe_2_O_4_ layers on glass substrates to evaluate differences arising from processing and form factor [20]. Veis and colleagues reported good agreement between experiment and calculations based on the permittivity tensor for rf-sputtered CuFe_2_O_4_ layers, with longitudinal Kerr rotation reaching up to ∼0.045° [179]. Likewise, Liskova-Jakubisova and coworkers examined 120 nm thick polycrystalline ZnFe_2_O_4_ films deposited on quartz by laser ablation and measured MOKE amplitudes up to ∼0.1°, which they contrasted with stronger effects in MgFe_2_O_4_ (≈1.5°) and Li_0.5_Fe_2.5_O_4_ (≈2.4°) [180].

A parallel thread in the literature is deliberate modification of spinel composition by doping or substitution to tune magneto-optical response. Zhou and colleagues investigated rare-earth substitutions in nanocrystalline thin films of CoFeMnO_4_ made by a sol-gel method, replacing a portion of atoms with Y^3+^, Lu^3+^, Yb^3+^, Tm^3+^, Tb^3+^, Sm^3+^ and La^3+^, and observed Kerr rotation increase from 0.65° for the undoped layer to 1.23° for CoFeMn_0.9_Tb_0.1_O_4_ [181]. In a related study, Avazpour et al. explored Nd and Pr substitution in cobalt ferrite thin films prepared by sol-gel synthesis and spin coating; they reported Kerr rotation up to ∼0.8° for polycrystalline unmodified films and found that Eu addition produced a clearer effect than Nd, with both substitutions producing a blue shift [182].

Taken together, these theoretical and experimental studies suggest that large Kerr responses in spinels depend sensitively on cation species, site occupancy, charge-transfer channels and film quality. For a broader perspective on these trends and on differences between polarized and natural-light responses in low- and high-magnetostriction spinels, see the review by Sukhorukov and colleagues [183].

In garnets, from a microscopic perspective, the off-diagonal permittivity depends on the energies of electronic transitions, their oscillator strengths, and the spin-orbit splitting of the excited states. Further improvement of outstanding properties of Y_3_Fe_5_O_12_ garnet by doping were extensively analyzed. Thin films of YIG were doped with cerium [184,185,186], bismuth [187], cerium and bismuth simultaneously [188], bismuth and gadolinium [167], terbium, dysprosium [189], gadolinium, gallium, aluminum [190], cobalt [191], manganese [192], and praseodymium [193].

Kehlberger et al. introduced cerium substitution in Y_3_Fe_5_O_12_ garnet layers fabricated by pulsed laser deposition on Gd_3_Ga_5_O_12_ with the thickness in the range of 96–112 ± 1 nm [184]. The authors report that the introduction of cerium resulted in doubling the of magneto-optic Kerr rotation at λ = 406 nm and a tenfold increase at λ = 635 nm. Moreover, they observed a strong contrast for the different magnetic domain orientations after doping.

Gizhevskiĭ and colleagues measured the influence of nanostructurization of Y_3_Fe_5_O_12_ nanoparticles. The material was synthesized by pyrolysis from nitrate precursors of the metals. After the heat treatment at 1000 °C for 3–5 h, the authors obtained amorphous powders with the average grain size of ≈1 μm [194]. The nanostructurization was realized by compressing the powder at up to 9 GPa and applying shear deformation by rotating one anvil with respect to the other at the rotation range of 0.3–1.0 rpm in air at room temperature. Although the authors report the magneto-optical effect and absorption spectra in the visible range are in agreement with the single crystals, they observed additional absorption bands at 2 and 3 μm. The obtained Kerr effect spectra have extrema corresponding to the single crystals but are smaller in magnitude and of a different shape. Their explanation is that the nanostructurization process introduces high density of point defects primarily due to the violation of the stoichiometry and the valence state of iron ions.

Tomita and others analyzed the influence of Au nanoparticles on optical properties of Y_3_Fe_5_O_12_ garnet films. The layers were prepare by rf sputtering simultaneously from Au and YIG targets on a quartz substrate [195]. The researchers checked three volume concentrations of gold in the YIG layers: 0, 1.7 and 10.9%. The thickness of the layers layers was approximately 200 (0%, 1.7%) and 500 nm (10.9%). The gold particles were of 12 nm diameter. The authors reported that Kerr rotation angles become negative in value in the region where the localized surface plasmon polariton resonance of the Au particles is located. This interesting observation indicates a possible coupling between the magneto-optic Kerr effect of YIG and the surface plasmon polariton even at as low content of gold as, at most, 10.9%. The authors explain this phenomenon by the high surface area of the 12 nm Au nanoparticles. In materials like YIG, charge-transfer transitions in the visible range contribute significantly to the MO response due to their high oscillator strength. In contrast, crystal-field (CF) transitions, typically involving intra-3d excitations, are parity-forbidden and thus exhibit much weaker contributions.

Murzina et al. fabricated films of YIG nanoparticles with the layer-by-layer deposition from a basic aqueous suspension containing 32 nm particles [196]. The authors reported a noticeable magnetic contrast of non-linear magneto-optical Kerr effect as a manifestation of the internal homodyne mechanism.

Magneto-optical Kerr effect is also observed in thin layers of garnets. Dolgova et al. fabricated a magnetophotonic crystal from a magnetic garnet spacer of Bi_0.1_Y_2.5_Fe_5_O_*x*_ (of half-wavelength thickness) located between SiO_2_ and Ta_2_O_5_ dielectric Bragg reflectors of quarter wavelength thickness [197]. The layers were rf sputtered on a glass substrate and annealed in air at 700 °C for 20 min. The authors used permanent FeNdB magnet applying dc-magnetic field of up to 2 kOe along the surface for the longitudinal and transversal configurations to observe nonlinear magneto-optical Kerr effect. The resulting angle of polarization rotation was 38° and 48° for the incidental angle of 30° and 15°, respectively.

Cai and colleagues predicted the giant MOKE inn orthorhombic BiNiO_3_ perovskite with the use of first-principle computations with the effect theoretically reaching up to 1.28° at 1.87 eV [198].

Based on their first-principle computations, Fan and colleagues stated that material made of chromium-based perovskite metal–organic framework should be multiferroic and magnetoelectric [199]. The authors found that the Kerr spectra changes significantly with the change of the ferroelectric polarization, which could result in the possibility of electric tuning of the Kerr angle. Moreover, reversing both the electric polarization and magnetization altogether changes the Kerr angle which may open the possibility for new electric-optical devices utilizing ferroelectric antiferromagnetic compounds.

Lu et al. performed first-principle density functional calculations to investigate electronic structure, magneto-optical effects, and topological properties of a cubic double perovskite Ba_2_NiOsO_6_ and its (111) (Ba_2_NiOsO_6_)_1_/(BaTiO_3_) monolayer superlattice [200]. They found that Ba_2_NiOsO_6_ exhibits strong magneto-optic effect (Kerr rotation up to 6°) because of enhanced intra-atomic exchange splitting of Os atoms caused by Ni 3d—Os 5d hybridization and the strong spin-orbit coupling on the osmium sites.

In another example of utilizing the power of density functional theory and first-principles calculations, Zu and colleagues explored the giant magneto-optical Kerr effect in Sr_2_CrWO_6_, Sr_2_CrReO_6_ and Sr_2_MoOsO_6_ double perovskites in the infrared to visible range [201]. They calculated that the maxima Kerr rotation for the corresponding compounds can reach up to −5.02, 2.56, and 4.45°.

Melnikov et al. reported a terahertz Kerr effect in CH_3_NH_3_PbBr_3_ perovskite single crystal induced by a nearly single-cycle terahertz pulse [202]. According to the authors, this effect is a composite of the Kerr effect in the inorganic lattice of the crystal, terahertz-induced transient alignment of CH_3_${\mathrm{NH}}_{3}^{+}$ cations and their coherent rotation in a Raman process.

A magneto-optical Kerr effect in hole-doped La_1−*x*_Sr_*x*_MnO_3_ and La_1−*x*_Sr_*x*_CoO_3_ with *x* in the range of 0–0.3 in perovskite crystals was described by Yamaguchi and colleagues [203]. The perovskites were grown via the floating-zone method from molten precursor oxides. The observed Kerr rotation was in the range of up to 10^−2^ degree for all compositions.

Fleischer and others produced material consisting of Mn_2_Ru_*x*_Ga in a cubic structure with a slight substrate-induced tetragonal distortion from the MgO substrate [204]. The material was co-sputtered from Ru and Mn_2_Ga targets on the MgO (magnetron-sputtered) film, and the ruthenium concentration was tested at 0.44 ≤x≤ 1.19. The authors proved that MOKE in zero-moment half metals persists even when the net magnetic moment crosses zero. The obtained Kerr rotation was in the range of 0.1°.

Hasanirokh and colleagues conducted numerical research on a (1 + 2)-dimensional graphene wormhole, which is a theoretical construct where a graphene sheet is curved and deformed into a wormhole-like structure [205]. The authors concluded that the orbital angular momentum, the radius of curvature, and wormhole coordinate can effectively control the linear magneto-optical Kerr effect.

### 4.4. Electro-Optical Kerr Effect in Materials

The first transparent ferroelectric ceramic, Pb(Zr,Ti)O_3_, was successfully produced in 1970 using hot-press sintering, a method that suppresses pore formation and enables a highly dense microstructure [206] Since then, transparent ferroelectric ceramics have attracted considerable attention because they combine desirable functional properties with practical advantages such as low fabrication cost, ease of shaping into complex geometries, and precise compositional tunability—benefits that are difficult to achieve with single crystals [206,207].

Analyzing epitaxial ferroelectric films of (Ba,Ca)(Ti,Zr)-O_3_ perovskite titanate, deposited by pulsed laser deposition of BCTZ targets synthesized by the solid-state reaction technique on single crystal SrTiO_3_ (100) substrate, Ion et al. reported strong electro-optical response in the form of Pockels and Kerr effects [208]. The authors indicated that the presence of both effects could be beneficial for photonic chips based on cascaded Pockels and Kerr nonlinear optical effects for quantum communication applications.

Salman and others synthesized the following composites based on perovskites: BaTiO_3_, 0.1 BiFeO_3_ + 0.7 BaTiO_3_ + 0.2 NdMnO_3_, 0.1 BiFeO_3_ + 0.7 BaTiO_3_ + 0.2 DyMnO_3_ and 0.1 BiFeO_3_ + 0.7 BaTiO_3_ + 0.2 Nd_0.5_Dy_0.5_MnO_3_ via sol-gel auto combustion and a solid-state reaction [209]. The researchers measured remanent polarization maximum polarization, recoverable energy density, and ferroelectric efficiency and chose 0.1 BiFeO_3_ + 0.7 BaTiO_3_ + 0.2 Nd_0.5_Dy_0.5_MnO_3_ composite as the most suitable candidate for energy storage applications out of the synthesized compounds. Moreover, they carried out Python modeling with ferroelectric data to observe the electro-optic Kerr effect in the form of a linear correlation between the dielectric constant and electric field in the compound.

Din et al. made a composite based on two perovskite phases and one spinel phase [9]. The perovskite phases were BiFeO_3_ (BFO) and PbZr_0.58_Ti_0.42_O_3_ (PZT), while the spinel was CoFe_2_O_4_ (CFO). The compounds were synthesized using the sol-gel technique (CFO), a hydrothermal reaction (BFO), and a solid-state reaction (PZT) with the use of ball-milling, which was also utilized to combine all three constituents of the composite. The final product, 0.9 [(1−x)BFO + *x*PZT] + 0.1 CFO (*x* = 0.0, 0.1, 0.2 and 0.3), exhibits magnetoelectric coupling and electro-optical Kerr effect and has potential applicability in energy harvesting and fast pulsating devices.

Huber and others extended a conventional one-dimensional optical into a two-dimensional optical Kerr spectroscopy to establish a unified origin of ultrafast Kerr responses in single crystal lead halide perovskites, in particular with the example of CsPbBr_3_ [210,211].

Van der Waals semiconductors are another interesting candidate to observe optical effects. Among them are compounds with the general formula MPX_3_, where M = V, Mn, Fe, Co or Ni and X = S or Se, particularly in a quasi-two-dimensional form [212]. Cheng and colleagues synthesized a quasi-2D MnPS_3_ single crystal via chemical vapor deposition [213] and investigated THz light-induced effects. They discovered that the material exhibits transient electro-optical Kerr effect and a value of nonlinear refractive index of approximately 13.1 × 10^−14^ cm^2^/W, which is an order of magnitude higher than typical bulk EO modulator materials.

### 4.5. Other Effects

The strength of the Kerr lens effect depends on the peak intensity of the radiation, making it particularly pronounced in pulsed laser systems. This principle is utilized in the construction of femtosecond Ti:sapphire lasers (also called Ti:sapphire oscillators), where the laser cavity exhibits lower losses during pulsed operation compared to continuous-wave operation.

Liu et al. reported the first diode-pumped Kerr-lens mode-locked femtosecond laser based on Yb:CaSrBaF_6_ single crystal [214]. The mode-locked laser has a peak wavelength of ≈1057 nm (FWHM ≈ 10.2 nm) with a pulse width of 123 fs at the maximum average output of 159 mW.

Lacerda and colleagues conducted calculations on the optical and magneto-optical properties in PbNiO_3_, PbCrO_3_ and PbMnO_3_ perovskites [215]. Their investigation on the connection between Kerr effects and excited electronic states in the compounds revealed that both optical and magneto-optical Kerr effects occur in them. Their coexistence is explained by the interaction between the material and incident radiation with characteristic wavelength creates an excited state which presents a different refractive index in regards to the ground state. Moreover, the states cause changes in the magnetic ordering because of the incident radiation.

In superconducting systems, the polar Kerr effect (PKE) serves as a sensitive probe for detecting time-reversal symmetry (TRS) breaking. This effect manifests as a nonreciprocal phase shift between left- and right-circularly polarized components of reflected light, acting as an optical signature of TRS violation. For instance, PKE measurements have revealed broken TRS in the superconducting phase of Sr_2_RuO_4_ [216].

### 4.6. Applications of Kerr Effects

Kerr effects are widely applied across various branches of technology and in a broad range of devices, including electro-optic light modulators, optical switches, telecommunication systems, sensors, and advanced photonic and display technologies. Owing to its sensitivity to external electric fields, the Kerr effect enables precise control and characterization of the optical properties of materials, such as field-induced birefringence. However, one of the most significant and scientifically compelling aspects of the Kerr effect lies in its application to fundamental studies of molecular properties. KE measurements provide valuable insight into molecular polarizability anisotropy, electronic structure, orientational dynamics, and intermolecular interactions. As a result, the KE has become an important spectroscopic tool in molecular physics and physical chemistry. Below, the most influential studies employing the Kerr effect for the investigation of molecular properties are presented.

For the MOKE, significant attention is given to applications in magnetic materials and spintronics, where the magneto-optical signal is used for the detection and characterization of dynamic magnetic textures and spin domains. Recent experimental studies show a robust Kerr response in noncollinear antiferromagnetic systems, enabling local probing of Berry curvature and supporting the development of antiferromagnetic spintronic devices with high thermal stability [136,217]. Additionally, organic–inorganic interfaces in magnetic thin films have been found to greatly enhance the MOKE signal through favorable orbital hybridization at molecule–metal interfaces, presenting new opportunities for high-sensitivity magnetic field sensors and magneto-optical memory elements [218,219]. Furthermore, advanced MOKE microscopy techniques with extreme antireflection conditions enable real-time imaging of sub-diffraction-scale magnetic domain dynamics, which is essential for understanding spin dynamics in next-generation magnetic memory technologies [220].

In recent years, the EOKR in molecular liquid crystals has been shown to exhibit significantly enhanced electro-optic coefficients through the incorporation of appropriate dopants, enabling the development of highly sensitive electro-optic modulators and nonlinear display components operating at low electric fields [221,222]. For example, liquid-crystal mixtures with higher dopant concentrations display a systematic increase in the Kerr constant, which directly improves the performance of light-modulating devices at reduced operating voltages [221,222,223]. Furthermore, molecularly engineered two-dimensional ferroelectric materials can achieve exceptionally high Kerr constants, allowing electro-optic devices to operate under ultralow electric fields while maintaining strong ultraviolet stability, paving the way for applications in large-area interactive displays and low-power optical switches [224,225].

In the realm of the optical Kerr effect, hybrid platforms that utilize thin-film materials like lithium niobate on insulator facilitate the creation of hybrid frequency combs characterized by high stability and wide spectral bandwidth [226]. This capability is crucial for telecommunications, precision spectroscopy, and quantum photonics, as well as for the advancement of high-performance frequency-comb lasers that do not rely on traditional bulk laser sources [227]. Additionally, molecular organic and hybrid materials, due to their significant electronic anisotropy, allow for the development of compact and energy-efficient nonlinear components in integrated photonics, where the precise manipulation of molecular structure directly influences the strength and effectiveness of nonlinear optical effects. These materials are also being actively explored for all-optical switching and for photonic elements with neuromorphic functionalities [228,229,230]. Table 2 summarizes the chemical compounds, transparency ranges, and Kerr effects.

## 5. The Future and Challenges of Kerr Effect Applications

Based on the optical systems presented in Section 3, a clear trend can be observed in the evolution of setups designed to measure the Kerr effect in various materials. These systems have progressed from simple configurations composed of a few basic optical components to sophisticated research tools. Regardless of the specific setup geometry, advances in technology—particularly in material quality—have significantly enhanced measurement accuracy and reduced light absorption losses. These improvements have, in turn, enabled the detection of increasingly weaker signals. One example of this progress is the development of polarizers. Their polarization efficiency has improved substantially, allowing for better-polarized output beams while minimizing absorption. Currently, researchers have improved optical setups by incorporating additional beam-shaping components such as quarter- and half-wave plates, mirrors, and diaphragms. A major milestone was the introduction of phase-sensitive amplifiers, which facilitate the detection of weak currents and help to extract meaningful signals from background noise.

Some research groups have focused on increasing the intensity of the magnetic field applied to samples—reaching up to several tens of tesla—to amplify the Kerr response. Another notable trend involves the integration of multiple phase-sensitive (lock-in) amplifiers within the setup to reduce noise and achieve higher measurement resolution.

Currently, optical systems for Kerr effect measurements can be classified into three main categories. The first category is compact optical systems for bulk sample measurements.

These systems are the most widely used and have undergone extensive modifications through the integration of additional measurement components and automation features. Most contemporary setups are equipped with data acquisition systems and digital measurement devices, allowing for automated control and analysis. A key goal is to develop fully automated systems with capabilities such as Wavelength tuning of the incident beam, Measurement of higher-order Kerr effects under strong magnetic field and light beam.

Such systems are relatively cost-effective and can be assembled in-house by many research groups. Their modular design enables customization to meet specific research needs and accommodate different types of materials. Both free-space- and fiber-based configurations are employed. Additionally, advanced interferometric techniques such as fiber-based, loop-less Sagnac interferometers and dual-beam setups are increasingly used. These allow for precise time delay control between the pump and probe beams via a delay stage, improving temporal resolution in pump-probe experiments.

The next category is confocal microscopy systems. Analysis evolved from the compact systems mentioned above; confocal microscopy setups are tailored for microscale surface measurements, enabling the study of nanostructures and domain visualization. Although they utilize similar core components such as balanced polarimeters and lock-in amplifiers, they also incorporate CCD camera. Recent advances in magneto-optical Kerr effect (MOKE) microscopy increasingly focus on hybrid and multimodal systems that extend its applicability to molecular-scale investigations. One important direction is the combination of Kerr microscopy with time-resolved pump–probe optical setups, enabling access to ultrafast spin and magnetization dynamics in molecular magnets and spin-crossover systems, where light-induced changes in molecular states directly affect magnetic response. Such configurations are particularly valuable for studying photo-switchable molecules and non-equilibrium spin phenomena.

Another significant development is near-field Kerr microscopy, often integrated with atomic force microscopy-based probes, which overcomes the diffraction limit of conventional optics. This approach allows localized probing of magneto-optical signals from molecular assemblies, self-assembled monolayers, or single-molecule magnets, where magnetic contrast is inherently weak and spatially confined. The enhanced spatial resolution is crucial for correlating the molecular arrangement with local magnetic anisotropy.

Kerr microscopy has also been combined with electrical transport and opto-electronic measurement platforms, allowing for simultaneous optical detection of magnetization and electrical readout. For molecular spintronic devices, this integration enables direct correlation between molecular magnetic states, charge transport, and spin-dependent effects induced by electric fields or currents. Such systems are essential for understanding spin injection and manipulation at molecule–electrode interfaces.

Finally, Kerr-based techniques are increasingly used in correlative approaches with vibrational spectroscopies, such as Raman or FT-IR microscopy. While often implemented sequentially rather than in a single instrument, this combination provides complementary information on molecular structure, bonding, and electronic states under external magnetic or electric fields. Together, these multi-modal strategies significantly broaden the role of Kerr microscopy from classical magnetic domain imaging toward comprehensive molecular-scale magneto-optical characterization. Other associated techniques include magnetic force microscopy and scanning tunneling microscopy or scanning electron microscopy with polarization analysis and an atomic force microscope. The advantages of these systems include improved signal filtering and significantly smaller optical spot sizes, which enhance spatial resolution. However, they are more complex and costly to construct.

The third category consists of systems utilizing synchrotron radiation, which are specifically designed for analyzing thin films and complex layered structures. Due to the nature of synchrotron light sources, only a limited number of dedicated measurement beamlines are available worldwide. These facilities provide unmatched sensitivity and resolution, making them essential for high-precision Kerr effect studies in advanced materials.

Kerr-effect instrumentation is not free of several technical and conceptual challenges that also define the most promising research directions. A major frontier is the development of ultrafast and broadband Kerr spectroscopy where femto- and picosecond pump-probe systems allow access to rapid spin and carrier dynamics. However, maintaining polarization intact, minimizing dispersion, and synchronizing pulses with sub-picosecond precision remain significant obstacles. Quantitative Kerr metrology would benefit from standardized calibration procedures and reference samples with certified rotation values, which are currently lacking. Modern Kerr analyses rely on density functional theory, magneto-optical conductivity tensor calculations and machine-learning-assisted data extraction. However, realistic simulations must incorporate defects, cation inversion and short-range order that are common in real materials. Data acquisition itself remains limited by instrumental drift, mechanical instabilities and laser noise. Future systems will likely rely on AI-assisted noise filtering and real-time signal separation.

Another approach involves miniaturization of on-chip Kerr sensors. This requires stable integrated polarizers, wave-plates, and polarization-preserving waveguides that can function over short optical paths without excessive noise.

Combining Kerr measurements with extreme conditions, like ultra-low temperatures, high pressures, or magnetic fields of several tens of tesla, is related with additional challenges in the form of optical access, vibration and unwanted Faraday rotation in cryogenic windows. Measurements on emerging materials such as two-dimensional magnets, van der Waals heterostructures, magnetic topological insulators, or plasmonic metasurfaces face additional difficulties which derive from small interaction volumes or laser-induced heating.

In summary, applications of the electric Kerr effect in modulators and digital light-processing devices, the optical Kerr effect in integrated photonics, and the magneto-optical Kerr effect in sensors and spintronic systems demonstrate that the molecular design of functional materials is a fundamental tool for optimizing the performance and functionality of next-generation technological devices.

In the future, electro-optic materials with high switching speeds—such as advanced nonlinear polymers and highly efficient light-modulating crystals—will be further developed. Significant progress is also expected in 2D materials, including graphene and transition-metal dichalcogenides, enabling greater miniaturization and higher performance of photonic control systems.

Finally, although synchrotron-based Kerr spectroscopy provides unmatched sensitivity and element specificity, access to beamlines is limited and sample preparation can be demanding. This motivates the development of tabletop high-harmonic-generation sources that may eventually deliver similar capabilities in laboratory-scale instruments.

## Figures and Tables

**Table 1:** Features of Kerr effect of various orders with relevance to MOKE and EOKE.

Feature	Kerr Effect—1st Order	Kerr Effect—2nd Order	Kerr Effect—3rd Order (Optical Kerr)	Higher-Order Kerr Effect (HOKE)
Type of Field	Static electric or magnetic field	Light + external electric field	High-intensity light (laser)	Ultra-high intensity light
Change (Δn)	$\propto E^{2}$ (EOKE), ∝M (MOKE linear)	$\propto E_{\mathrm{opt}}\cdot E_{\mathrm{ext}}$ (EOKE), possible weak nonlinear MOKE	$\propto I={\left|E\right|}^{2}$ (EOKE), some nonlinear MOKE reported	$\propto I^{2},I^{3},\ldots$ (mainly EOKE)
Order of nonlinearity	Classical quadratic (EOKE), linear or weakly nonlinear (MOKE)	2nd order (mainly EOKE)	3rd order (mainly EOKE), some nonlinear MOKE	5th, 7th, … (mainly EOKE)
Susceptibility	Mixed or $\chi^{\left(1\right)}$ (EOKE), linear magneto-optic tensor (MOKE)	$\chi^{\left(2\right)}$ (EOKE), some second harmonic magneto-optic effects (MOKE)	$\chi^{\left(3\right)}$ (EOKE), nonlinear magneto-optic responses possible	$\chi^{\left(5\right)},\chi^{\left(7\right)},\ldots$ (mainly EOKE)
Material requirements	Isotropic dielectric (EOKE), ferromagnetic metals or multilayers (MOKE)	Non-centrosymmetric crystals (EOKE)	Any material (glass, liquids) (EOKE)	Any material, extreme conditions (EOKE)
Light intensity	No (EOKE static), No or low (MOKE)	Yes (EOKE), low or moderate (MOKE nonlinear)	Yes (intense, EOKE), some nonlinear MOKE effects require high intensity	Yes (ultra-intense, mainly EOKE)
Physical effect	Electrically induced birefringence (EOKE), magnetization-induced polarization rotation (MOKE)	Electro-optic mixing, SHG (EOKE), magneto-optic SHG possible (MOKE)	Self-focusing, SPM, solitons (EOKE), nonlinear magneto-optic dynamics reported	Spectral broadening, nonlinear phase shifts (mainly EOKE)
Applications	Kerr cells, electro-optic modulators (EOKE), magneto-optic sensors (MOKE)	Frequency doubling, Pockels effect (EOKE), magneto-optic SHG imaging (MOKE)	Nonlinear optics, supercontinuum generation (EOKE), ultrafast magnetization dynamics (MOKE)	High-field physics, attosecond science (mainly EOKE)

Note: MOKE is primarily a magnetization-induced linear or weakly nonlinear optical phenomenon, while EOKE covers classical and higher-order nonlinear optical Kerr effects induced by electric fields or intense light. Some nonlinear magneto-optical effects can be seen as higher-order MOKE phenomena but are less commonly classified in this scheme.

**Table 2:** Summarized parameters of materials exhibiting MOKE and EOKE effects.

Compound	Type	Transparency Range	MOKE	EOKE	Ref.
CeSb, CsSb_*x*_Te_*1−x*_	rock-salt	–	>14°, up to $90^{{}^{\circ}}$	–	[173,174]
FeCr_2_O_4_	spinel	–	strong (qualitative)	–	[175]
CoCr_2_O_4_	spinel	∼0.78 eV (1.55 μm)	≈12°	–	[175]
CoFe_2_O_4_	spinel	–	large (DFT)	–	[176]
NiFe_2_O_4_	spinel	–	∼3× lower than CoFe_2_O_4_	–	[177]
CoFe_2−*x*_Al_*x*_O_4_	spinel	0.6–5.5 eV	composition dependent	–	[178]
Co_*x*_Fe_3−*x*_O_4_	spinel	0.6–5.5 eV	composition dependent	–	[178]
CuFe_2_O_4_	spinel	–	up to $0.045^{{}^{\circ}}$	–	[179]
ZnFe_2_O_4_	spinel	–	up to $0.1^{{}^{\circ}}$	–	[180]
MgFe_2_O_4_	spinel	–	≈1.5°	–	[180]
Li_0.5_Fe_2.5_O_4_	spinel	–	≈2.4°	–	[180]
CoFeMnO_4_ (RE-doped)	spinel	–	$0.65^{{}^{\circ}}\to 1.23^{{}^{\circ}}$	–	[181]
Co_*x*_RE_(1−*x*)_Fe_2_O_4_	spinel	–	$1.7^{{}^{\circ}}$	–	[182]
Hg_(1−*x*)_Cd_*x*_Cr_2_Se_4_	spinel	IR	$0.5^{{}^{\circ}}$	–	[183]
Y_3_Fe_5_O_12_ (YIG)	garnet	visible–NIR	enhanced by doping	–	[184]
Ce:YIG, RE:YIG, YIG	garnet	406–635 nm	2× to 10× increase	–	[184,185,186,187,188,189,190,191,192,193]
Nanostructured YIG	garnet	visible; add. bands at 2–3 μm	reduced vs. single crystal	–	[194]
Au–YIG	garnet composite	visible	sign reversal	–	[195]
YIG nanoparticles	garnet	–	nonlinear MOKE	–	[196]
Bi_0.1_Y_2.5_Fe_5_O_*x*_	garnet	visible	$38^{{}^{\circ}}$–$48^{{}^{\circ}}$ (nonlinear)	–	[197]
BiNiO_3_	perovskite	∼1.87 eV	up to $1.28^{{}^{\circ}}$ (theory)	–	[198]
Ba_2_NiOsO_6_	double perovskite	–	up to 6°	–	[200]
Sr_2_CrWO_6_	double perovskite	IR–visible	up to $-5.02^{{}^{\circ}}$	–	[201]
CH_3_NH_3_PbBr_3_	perovskite	THz	–	transient Kerr	[202]
La_1−*x*_Sr_*x*_MnO_3_	perovskite	–	up to $10^{-2}$°	–	[203]
Mn_2_Ru_*x*_Ga	Heusler-like	–	≈0.1°	–	[204]
Graphene wormhole	2D carbon	–	tunable (theory)	–	[205]
Pb(Zr,Ti)O_3_ (PZT)	perovskite	transparent	–	EO Kerr	[206,207]
(Ba,Ca)(Ti,Zr)O_3_ (BCTZ)	perovskite	–	–	strong (Pockels + Kerr)	[208]
BaTiO_3_	perovskite	–	–	EO Kerr (composite)	[209]
0.1 BiFeO_3_ + 0.7 BaTiO_3_ + 0.2 NdMnO_3_	perovskite composite	–	–	EO Kerr (modeled)	[209]
0.1 BiFeO_3_ + 0.7 BaTiO_3_ + 0.2 DyMnO_3_	perovskite composite	–	–	EO Kerr (modeled)	[209]
0.1 BiFeO_3_ + 0.7 BaTiO_3_ + 0.2 Nd_0.5_Dy_0.5_MnO_3_	perovskite composite	–	–	EO Kerr (linear corr.)	[209]
0.9[BiFeO_3_ + xPbZr_0.58_Ti_0.42_O_3_] + 0.1 CoFe_2_O_4_	perovskite/spinel composite	–	–	EO Kerr	[9]
CsPbBr_3_	perovskite	–	–	ultrafast Kerr	[210,211]
MnPS_3_	layered vdW	THz (VIS)	–	$n_{2}\approx 13.1\times 10^{-14}$ cm^2^/W	[212,213]
Yb:CaSrBaF_6_	fluoride crystal	∼1057 nm	Kerr-lens	–	[214]
PbNiO_3_	perovskite	–	optical + MO Kerr	–	[215]
PbCrO_3_	perovskite	–	optical + MO Kerr	–	[215]
PbMnO_3_	perovskite	–	optical + MO Kerr	–	[215]
Sr_2_RuO_4_	layered perovskite	–	polar Kerr (TRS)	–	[216]

Note: for 0.9[BiFeO_3_ + xPbZr_0.58_Ti_0.42_O_3_] + 0.1 CoFe_2_O_4_, x = 0.0, 0.1, 0.2 and 0.3 (four compositions).

## Data Availability

No new data were created or analyzed in this study. Data sharing is not applicable to this article.

## Abbreviations

The following abbreviations are used in this manuscript:

ALON	commercial name of aluminum oxynitride
MOKE	magneto-optical Kerr effect
EOKE	electro-optical Kerr effect
PKE	polar Kerr effect
TRS	time-reversal symmetry
RCP	right-circularly polarized
LCP	left-circularly polarized
DC	direct current
AC	alternating current
PMT	photomultiplier tube
QWP	quarter-wave plate
HWP	half-wave plate
EOM	electro-optic modulator
PEEM	photoemission electron microscope
NPCF	nonlinear photonic-crystal fiber
CCD	charge-coupled device
